# Regulation of *nrf* operon expression in pathogenic enteric bacteria: sequence divergence reveals new regulatory complexity

**DOI:** 10.1111/mmi.13647

**Published:** 2017-03-01

**Authors:** Rita E. Godfrey, David J. Lee, Stephen J. W. Busby, Douglas F. Browning

**Affiliations:** ^1^ Institute of Microbiology and Infection, School of BiosciencesUniversity of BirminghamBirminghamB15 2TTUK; ^2^ Department of Life Sciences, School of Health SciencesBirmingham City UniversityBirminghamB15 3TNUK

## Abstract

The *Escherichia coli* K‐12 *nrf* operon encodes a periplasmic nitrite reductase, the expression of which is driven from a single promoter, *pnrf*. Expression from *pnrf* is activated by the FNR transcription factor in response to anaerobiosis and further increased in response to nitrite by the response regulator proteins, NarL and NarP. FNR‐dependent transcription is suppressed by the binding of two nucleoid associated proteins, IHF and Fis. As Fis levels increase in cells grown in rich medium, the positioning of its binding site, overlapping the promoter −10 element, ensures that *pnrf* is sharply repressed. Here, we investigate the expression of the *nrf* operon promoter from various pathogenic enteric bacteria. We show that *pnrf* from enterohaemorrhagic *E. coli* is more active than its K‐12 counterpart, exhibits substantial FNR‐independent activity and is insensitive to nutrient quality, due to an improved −10 element. We also demonstrate that the *Salmonella enterica* serovar Typhimurium core promoter is more active than previously thought, due to differences around the transcription start site, and that its expression is repressed by downstream sequences. We identify the CsrA RNA binding protein as being responsible for this, and show that CsrA differentially regulates the *E. coli* K‐12 and *Salmonella nrf* operons.

## Introduction

The *Escherichia coli* K‐12 *nrf* operon encodes the NrfA periplasmic formate‐dependent nitrite reductase, which is responsible for reducing nitrite to ammonium ions to support bacterial growth under anaerobic conditions (Darwin *et al*., 1993). In addition to its role in anaerobic respiration, the NrfA nitrite reductase can also reduce the toxic molecule nitric oxide (NO) and contributes to the ability of *E. coli* and *Salmonella enteric* serovar Typhimurium to detoxify NO anaerobically (Poock *et al*., 2002; Gilberthorpe and Poole, 2008; Mills *et al*., 2008; van Wonderen *et al*., 2008). As enteric bacteria are exposed to both nitrite and NO, during their transition through the mammalian gastrointestinal track, this makes the NrfA nitrite reductase an important enzyme in the anaerobic environment of the gut.

**Figure 1:**
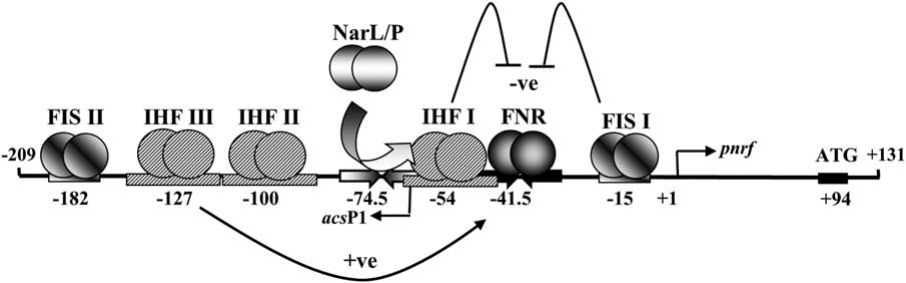
Organization of the *E. coli* K‐12 *pnrf53* promoter fragment. The figure shows a schematic representation of the *E. coli* K‐12 *pnrf53* promoter fragment and the important elements involved in its regulation. All numbering is in relation to the transcription start of *pnrf* (+1). The FNR and NarL/NarP binding sites are represented by inverted arrows, whilst the IHF and Fis binding sites are depicted by boxes. The central base pair of each DNA binding site is given, the transcription start site is indicated by an arrow and the location of the *nrfA* ATG initiation codon is shown. Expression from *pnrf* is completely dependent on FNR‐dependent activation, which is repressed (−ve) by IHF and Fis binding to IHF I and Fis I, respectively, and stimulated (+ve) by IHF binding to IHF III. NarL/NarP counteract the repressive effects of IHF, bound to IHF I, by displacing IHF from the promoter region. The location of the weak *acsP1* promoter is indicated by an arrow (Browning *et al*., 2002, 2005).

**Figure 2:**
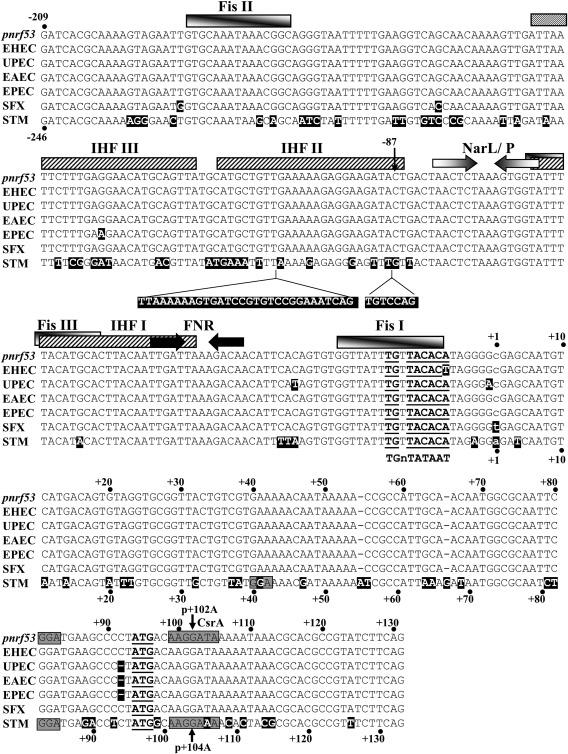
Alignment of the *nrf* promoter sequences from different enteric bacteria. The figure shows the sequence of the *E. coli* K‐12 *pnrf53* fragment from positions −209 to +131, aligned with the *nrf* promoter regions from EHEC, UPEC, EAEC, EPEC, *S. flexneri* (SFX) and *S. enterica* serovar Typhimurium (STM). The location of the transcription start site for *pnrf53* is indicated by lower case text. The location of FNR and NarL/NarP binding sites are represented by inverted arrows, whilst IHF and Fis binding sites are depicted by boxes. The CsrA binding sequences and GGA motifs in the *E. coli* K‐12 and *S. enterica* serovar Typhimurium leader sequences are highlighted by grey boxes. The insertion of sequences within the upstream promoter region of *S. enterica* serovar Typhimurium promoter is indicated. Differences between the *pnrf53* fragment and other promoters are highlighted in black. The extended −10 consensus sequence (TGnTATAAT) is aligned with the *pnrf* −10 promoter elements (Browning and Busby, 2016) and the location of the p + 102A and p + 104A substitutions, introduced into the *pnrf53* and *pnrf53* STM promoter fragments, respectively, is indicated by an arrow.

Transcription of the *E. coli* K‐12 *nrf* operon is driven from a single promoter (*pnrf*) and expression is induced by the global transcription activator protein, FNR, in the absence of oxygen (Page *et al*., 1990; Tyson *et al*., 1994). FNR binding to a single DNA site, centred at position −41.5 (i.e., between positions −41 and −42 relative to the transcription start site, +1), is sufficient for maximal *pnrf* induction (Tyson *et al*., 1994; Browning *et al*., 2002). However, FNR‐dependent activation is suppressed by the binding of two nucleoid‐associated factors, IHF (integration host factor) and Fis (factor for inversion stimulation). The *nrf* promoter contains three DNA sites for IHF (IHF I to III) and three DNA sites for Fis (Fis I to III) (see Figs 1 and 2) (Browning *et al*., 2002; Browning *et al*., 2005; Browning *et al*., 2006). Binding of IHF to IHF I and Fis to Fis I both repress FNR‐dependent transcription, whilst the occupancy of IHF III has a stimulatory effect (Browning *et al*., 2002; Browning *et al*., 2005; Browning *et al*., 2006) (Fig. 1). The *nrf* promoter is also regulated in response to nitrite and nitrate ions by the two homologous response regulators, NarL and NarP (Tyson *et al*., 1994; Darwin *et al*., 1997; Wang and Gunsalus, 2000). Both NarL and NarP bind to the same site positioned at −74.5 and their association with *pnrf* displaces IHF from IHF I, resulting in nitrite‐dependent activation and maximal *pnrf* expression (Fig. 1) (Browning *et al*., 2002; Browning *et al*., 2005; Browning *et al*., 2006). In addition, expression from *pnrf* is repressed when cells are grown in rich medium (Page *et al*., 1990; Tyson *et al*., 1997). This repression is mediated by Fis binding to Fis I. As the cellular concentration of Fis surges under nutrient rich conditions, this results in greater occupancy of Fis I, shutting down *pnrf* expression irrespective of other environmental cues (Ball *et al*., 1992; Browning *et al*., 2005).

During their evolution, bacterial pathogens are exposed to the particular environmental conditions within their host organism. Over time, their genomes accumulate mutations, many of which will have no effect, whilst others can change protein function or gene regulation. To understand how this process has shaped the expression of the *nrf* operon, we have examined the *nrf* operon promoters from a number of different enteric pathogens (Fig. 2), particularly focusing on the enterohaemorrhagic *E. coli* (EHEC) and *Salmonella enteric* serovar Typhimurium promoters. Using this approach, we have uncovered differences in the regulatory strategies used at these promoters in these organisms and identify the global regulator, CsrA, as an additional regulator of this complex operon in enteric bacteria.

## Results

### Analysis of *nrf* operon promoters from pathogenic *E. coli* and *Salmonella enterica* strains

**Figure 3:**
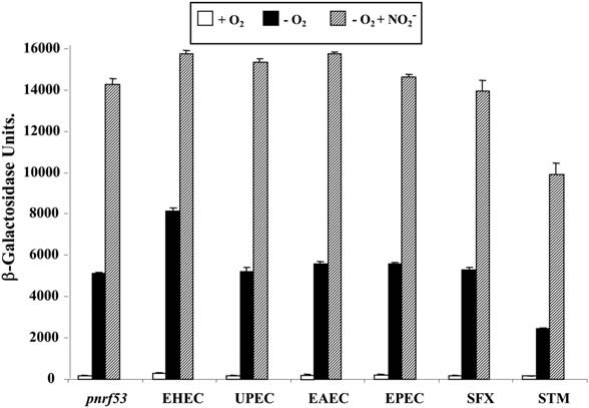
Expression of *nrf* promoters from different enteric bacteria in strain JBC387. The figure shows the β‐galactosidase activities of wild‐type JCB387 cells carrying pRW50, containing *pnrf53* promoter fragments from various enteric bacteria (see Fig. 2). Cells were grown aerobically and anaerobically in minimal salts medium and where indicated 2.5 mM sodium nitrite was added. β‐galactosidase activities are expressed as nmol of ONPG hydrolysed min^−1^ mg^−1^ dry cell mass, each activity is the average of three independent determinations and standard deviations are shown.

Previously, we generated the *E. coli* K‐12 *pnrf53* promoter fragment, which carries the *nrf* promoter sequences from −209 upstream of the transcription start site (+1) to +131 downstream (Figs 1 and 2). This fragment contains all the necessary DNA sequence required for anaerobic and nitrite induction (Tyson *et al*., 1994). The alignment of *pnrf*53 DNA sequence with the corresponding *nrf* operon sequences from different enteric pathogens indicated that there are some base pair differences in the transcription factor binding sites, the core promoter regions and translational initiation signals at many of the promoters, with sequence differences being particularly extensive for the *S. enterica* serovar Typhimurium promoter (Fig. 2). As these differences could affect the expression of the *nrf* operon in these bacteria, we generated similar *pnrf53* promoter fragments for EHEC, uropathogenic *E. coli* (UPEC), enteroaggregative *E. coli* (EAEC), enteropathogenic *E. coli* (EPEC), *Shigella flexneri* and *S. enterica* serovar Typhimurium (Fig. 2 and Supporting Information Table S1) (Browning *et al*., 2006). All fragments were cloned into the low copy number *lac* expression vector pRW50 (Lodge *et al*., 1992), to generate *lacZ* transcriptional fusions, and transformed into our wild‐type Δ*lac E. coli* K‐12 strain, JCB387. The expression of β‐galactosidase in JCB387 cells, carrying each promoter, was then determined when cultures were grown in minimal medium aerobically, anaerobically and anaerobically in the presence of nitrite. Results in Fig. 3 show that expression from all *pnrf* derivatives was induced by anaerobiosis and further increased in the presence of nitrite. Most *pnrf53* derivatives displayed similar expression levels to the *E. coli* K‐12 promoter, however, the EHEC promoter, *pnrf53* EHEC, was more active anaerobically, whilst, the *S. enterica* serovar Typhimurium promoter (*pnrf53* STM) was less active, as previously demonstrated (Browning *et al*., 2006; Browning *et al*., 2010). As the *pnrf53* EHEC promoter possesses a single base pair difference in its −10 promoter element (TATACT) (Fig. 2), which improves its resemblance to the −10 consensus sequence (TATAAT) (Browning and Busby, 2016) and is a considerable distance from the FNR biding site, we examined whether expression was still completely dependent on FNR, by determining the promoter activity of constructs in the Δ*fnr* strains, JRG1728 and JCB387 Δ*fnr*. Results in Table 1 show that, whilst the majority of *pnrf53* derivatives possessed little promoter activity in JRG1728, the *pnrf53* EHEC promoter displayed considerable FNR‐independent activity in both JRG1728 and JCB387 Δ*fnr*. In support of this Western blots, using anti‐NrfA antiserum and whole cell lysates from aerobically grown cells, detected considerably more NrfA protein in EHEC strain EDL933 than in the *E. coli* K‐12 strains RK4353 and MG1655 (Supporting Information Fig. S1). Thus, the improvement of the *pnrf* EHEC −10 element allows some NrfA expression to occur in the absence of FNR and in the presence of oxygen. Note that amino acid sequences of the EHEC FNR, Fis and various RNA polymerase subunits are either identical or extremely similar to those of *E. coli* K‐12 (Supporting Information Fig. S2).

**Table 1:** Expression of *nrf* promoters from different enteric bacteria in JRG1728 and JCB387 Δ*fnr* strains.

		β‐Galactosidase activity^a^	
Promoter^b^	JCB387	JRG1728	JCB387 Δ*fnr*
*pnrf53*	5119 ± 46	54 ± 2	36 ± 1
*pnrf53* EHEC	8140 ± 177	412 ± 15	296 ± 13
*pnrf53* UPEC	5200 ± 216	71 ± 3	nd^c^
*pnrf53* EAEC	5568 ± 142	51 ± 2	nd
*pnrf53* EPEC	5564 ± 72	55 ± 1	nd
*pnrf53* SFX	5298 ± 112	65 ± 6	nd
*pnrf53* STM	2435 ± 36	69 ± 1	55 ± 1

**a.** β‐galactosidase activities were measured in the JCB387 and two Δ*fnr* stains, JRG1728 and JCB387 Δ*fnr*, carrying pRW50 containing different *pnrf53* fragments. Cells were grown anaerobically in minimal salts medium and β‐galactosidase activities are expressed as nmol of ONPG hydrolysed min^−1^ mg^−1^ dry cell mass. Each activity is the average of three independent determinations and standard deviations are shown.

**b.** The first column lists the *pnrf53* fragments used.

**c.** nd: not determined.

### The EHEC *pnrf* promoter is insensitive to nutrient quality

The *E. coli* K‐12 *nrf* promoter is repressed in rich medium, being down‐regulated by Fis binding to Fis I (Page *et al*., 1990; Tyson *et al*., 1997; Browning *et al*., 2005). To determine if the *pnrf* EHEC promoter was similarly regulated, we examined its promoter activity in the *narL narP* strain JCB3884 in minimal and rich media. Note that strain JCB3884 was used in this experiment to remove any effects that NarL or NarP activation have on promoter activity. Results in Table 2 show that expression from the *pnrf53* and *pnrf53* STM promoter fragments was similarly repressed in rich medium growth conditions (5.8‐ and 6.7‐fold respectively). However, expression from the *pnrf53* EHEC promoter fragment was relatively insensitive to medium composition, showing only a 2.2‐fold decrease. Thus, we conclude that improvement of the −10 element makes the EHEC promoter less sensitive to nutrient quality.

**Table 2:** Repression of *pnrf* promoters from different enteric bacteria in rich medium.

	β‐Galactosidase activity^a^		
Promoter^b^	Minimal medium	Rich medium	Ratio^c^
*pnrf53*	3254 ± 320	562 ± 19	5.8
*pnrf53* STM	1121 ± 107	168 ± 5	6.7
*pnrf53* EHEC	4766 ± 327	2146 ± 153	2.2

**a.** β‐galactosidase activities were measured in the *narL narP* strain JCB3884, carrying pRW50 containing different *pnrf53* promoter fragments. Cells were grown anaerobically in either minimal salts medium or rich medium (Lennox broth plus 0.4% glucose). β‐galactosidase activities are expressed as nmol of ONPG hydrolysed min^−1^ mg^−1^ dry cell mass, each activity is the average of three independent determinations and standard deviations are shown.

**b.** The first column lists the *pnrf53* fragments used.

**c.** The ratio column indicates the fold repression for each promoter in rich medium.

**Figure 4:**
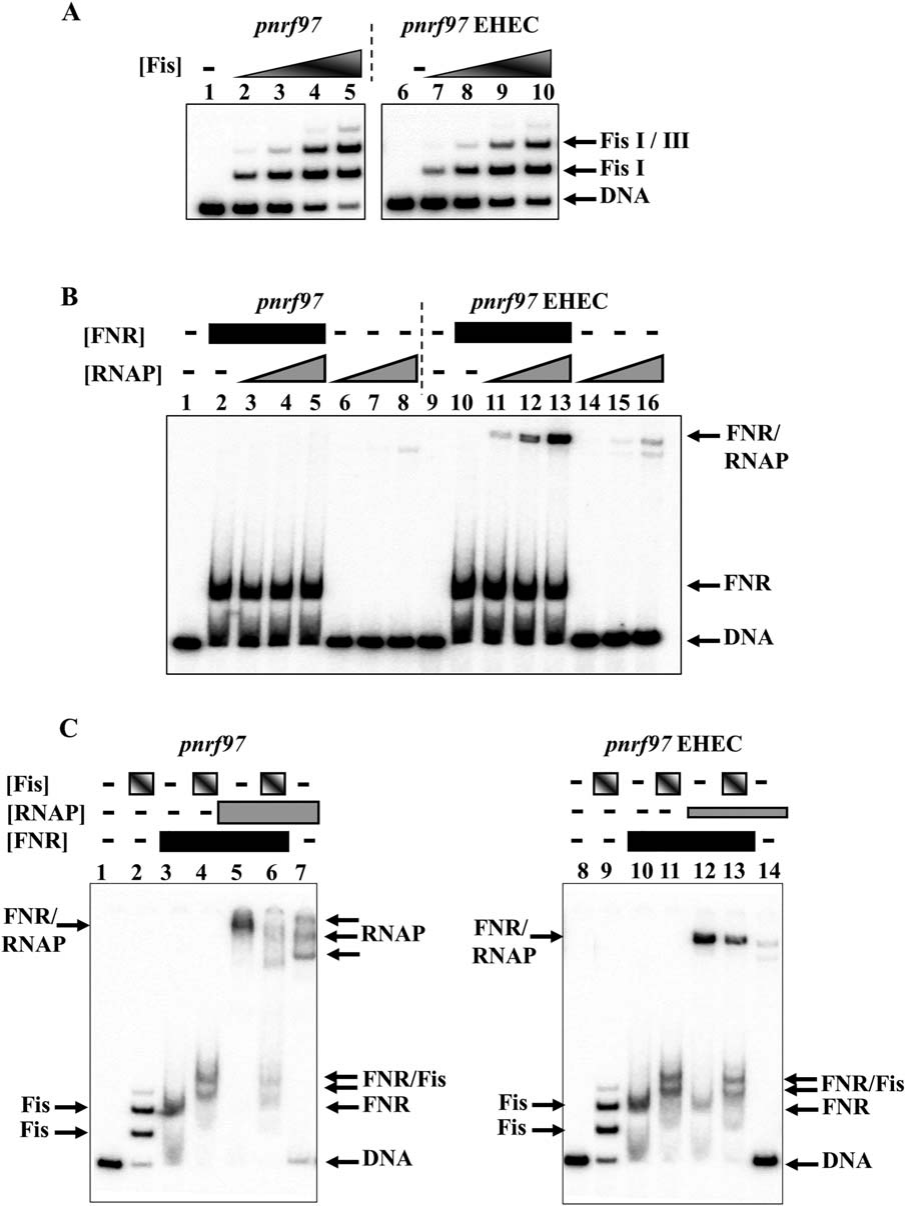
Gel retardation assays using *pnrf97* promoter fragments. The figure shows gel retardation assays of *pnrf97* fragments from *E. coli* K‐12 and EHEC, with purified Fis, FNR DA154 and RNA polymerase. A. End‐labelled *pnrf97* fragments were incubated with increasing concentrations of purified Fis protein: lanes 1–5, *pnrf97* EcoRI‐HindIII fragment; lanes, 6–10, *pnrf97* EHEC EcoRI‐HindIII fragment. The concentration of Fis protein in each reaction was: lanes 1 and 6, no protein; lanes 2 and 7, 50 nM; lanes 3 and 8, 100 nM; lanes 4 and 9, 150 nM; lanes 5 and 10, 200 nM. B. End‐labelled *pnrf97* fragments were incubated with purified FNR DA154 and increasing concentrations of purified RNA polymerase: lanes 1–8, *pnrf97* EcoRI‐HindIII fragment; lanes 9–16, *pnrf97* EHEC EcoRI‐HindIII fragment. The concentration of FNR protein in each reaction was: lanes 1, 6–8, 9 and 14–16, no protein; lanes 2–5 and 10–13, 2.7 µM. The concentration of RNA polymerase in each reaction was: lanes 1, 2, 9 and 10, no protein; lanes 3, 6, 11 and 14, 25 nM; lanes 4, 7, 12 and 15, 50 nM; lanes 5, 8, 13 and 16, 100 nM. C. End‐labelled *pnrf97* fragments were incubated with purified Fis, FNR DA154 and RNA polymerase: lanes 1–7, *pnrf97* EcoRI‐HindIII fragment; lanes 8–14, *pnrf97* EHEC EcoRI‐HindIII fragment. The concentration of Fis was: lanes 1, 3, 5, 7, 8, 10, 12 and 14, no protein; lanes 2, 4, 6, 9, 11 and 13, 200 nM. The concentration of RNA polymerase in each reaction was: lanes 1–4 and 8–11, no protein; lanes 5–7, 500 nM; lanes 12–14, 200 nM. The concentration of FNR protein in each reaction was: lanes 1, 2, 7–9 and 14, no protein; lanes 3–6 and 10–13, 2.7 µM.

**Figure 5:**
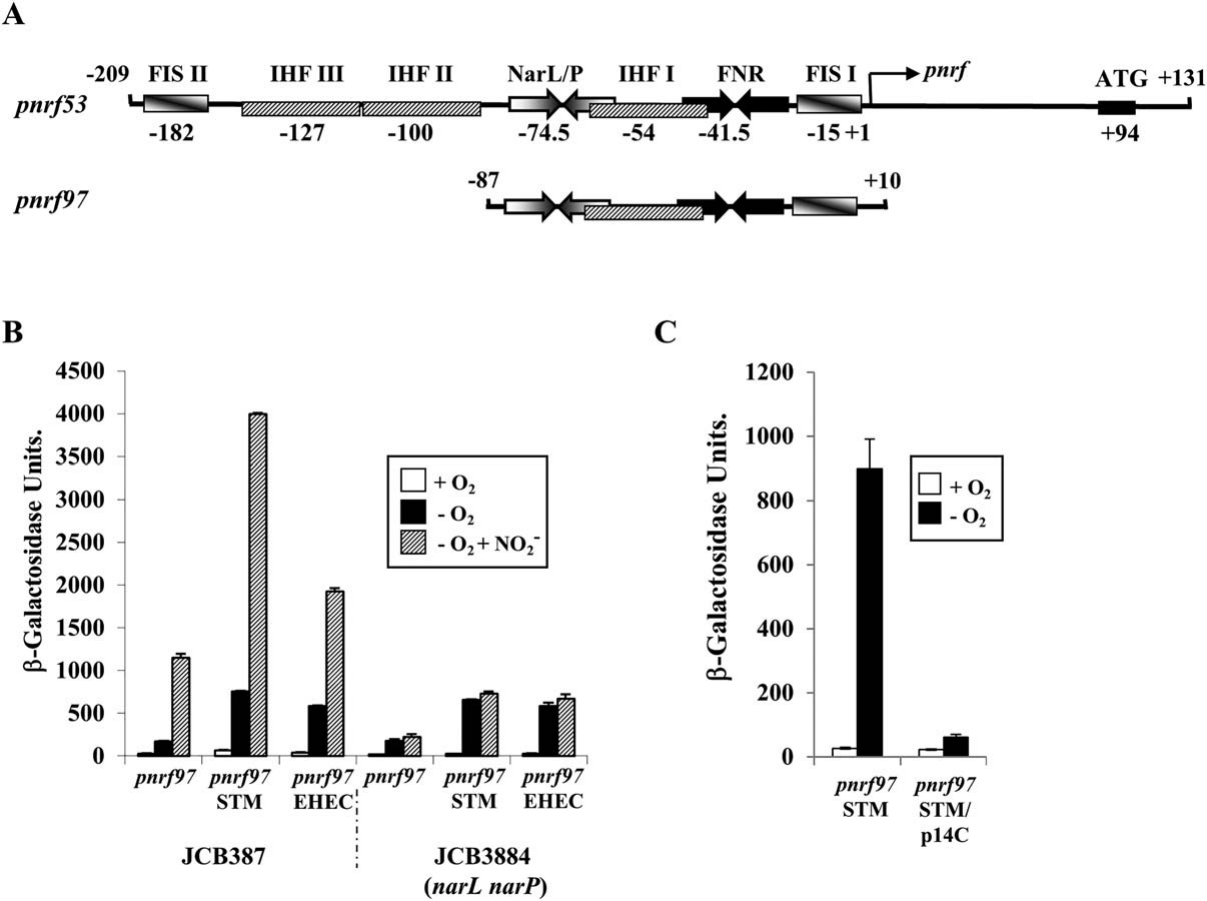
Expression of *pnrf97* promoter fragments from different enteric bacteria. A. The panel shows a schematic representation of the *E. coli* K‐12 *pnrf53* and *pnr97* promoter fragments. The location of FNR and NarL/NarP binding sites are represented by inverted arrows, whilst IHF and Fis binding sites are depicted by boxes. B. The panel shows the β‐galactosidase activities of wild‐type JCB387 and JCB3884 (*narL narP*) cells carrying pRW224, containing *pnrf97* promoter fragments (sequences −87 to +10) from *E. coli* K‐12, *S. enteric* serovar Typhimurium and EHEC (see Fig. 2). Cells were grown aerobically and anaerobically in minimal salts medium and where indicated 2.5 mM sodium nitrite was added. C. The panel shows the β‐galactosidase activities of JCB3884 (*narL narP*) cells carrying pRW224, containing either the *pnrf97* STM or the *pnrf97* STM/p14C promoter fragments (sequences −87 to +10). The p14C mutation introduces a point mutation at position −14 in *pnrf97* STM to disrupt the extended −10 promoter motif. Cells were grown aerobically and anaerobically in minimal salts medium. β‐galactosidase activities are expressed as nmol of ONPG hydrolysed min^−1^ mg^−1^ dry cell mass, each activity is the average of three independent determinations and standard deviations are shown.

Previously, we demonstrated that mutations that disrupt Fis binding to Fis I relieved repression under nutrient rich conditions (Browning *et al*., 2005). As this −10 element base change in the EHEC promoter lies just outside the Fis I binding site (Fig. 2), we used gel retardation assays to examine the binding of Fis to the *E. coli* K‐12 and EHEC *pnrf97* promoter fragments, which both carry *pnrf* DNA sequences from −87 to +10 (Figs 4A and 5A). Results in Fig. 4A show that both *pnrf97* fragments bound purified Fis similarly, with Fis I being occupied first and lower affinity Fis sites at higher concentrations, as previously observed in gel retardation and DNase I footprinting experiments (Browning *et al*., 2002, 2005). Thus, we conclude that differential Fis binding is not the reason why the EHEC *nrf* promoter can bypass repression under nutrient rich growth conditions.

As it is likely that the improvement of the EHEC *pnrf* −10 element is responsible for this alteration in regulation, we examined the binding of purified FNR and RNA polymerase to each *pnrf97* promoter fragment. Note that in this experiment FNR carries the DA154 substitution, which renders FNR active under aerobic conditions (Wing *et al*., 2000). As expected, FNR bound to each fragment, producing a single shifted species (Fig. 4B). The inclusion of 25 to 100 nM RNA polymerase resulted in super‐shifted RNA polymerase/FNR/DNA complexes for the *pnrf97* EHEC fragment (Fig. 4B; lanes, 11 to 13). However, similar complexes were not detected at these concentrations of RNA polymerase for the *E. coli* K‐12 *pnrf97* fragment (Fig. 4B) and were only observed at high concentrations of 500 nM (Fig. 4C; lane 5). Thus, as expected, RNA polymerase binds more strongly to the EHEC *pnrf* promoter in the presence of FNR. To investigate the effect of Fis on these complexes, we first pre‐incubated end‐labelled *pnrf97* fragments with purified Fis and/or FNR before adding RNA polymerase. Note that for the *E. coli* K‐12 *pnrf97* fragment RNA polymerase was used at a concentration of 500 nM, whilst for *pnrf97* EHEC a concentration of 200 nM was used. Results detailed in Fig. 4C, confirm that Fis and FNR can bind simultaneously to each promoter fragment (lanes, 4 and 11) and that FNR facilitates the binding of RNA polymerase (lanes, 5 and 12). When the *E. coli* K‐12 *pnrf97* promoter fragment was pre‐incubated with Fis and FNR and then challenged with RNA polymerase, we were able to detect super‐shifted species (lane, 6). However, the diffuse nature of these complexes suggests that they are unstable during electrophoresis and that Fis interferes with RNA polymerase binding. Conversely, for the *pnrf97* EHEC fragment, stable RNA polymerase‐containing complexes were detected when both FNR and Fis were present in the reaction mix (lane, 13), indicating that Fis has less effect on the ability of RNA polymerase to bind to the EHEC promoter. Thus, we conclude that the EHEC *pnrf* promoter can bypass this repression as its improved −10 element allows RNA polymerase to out compete Fis when binding at the EHEC *nrf* promoter.

We note that for the *E. coli* K‐12 *pnrf97* promoter fragment, RNA polymerase shifted species were observed in the absence of FNR (Fig. 4C: lane 7). The *pnrf97* fragment contains the weak divergent *acsP1* promoter (Fig. 1), which is totally repressed by FNR binding (Browning *et al*., 2002, 2005) and, therefore, the complexes observed are due to occupation of the *acs*P1promoter at the higher RNA polymerase concentrations used for this fragment. Note that *in vitro* transcription experiments confirmed that FNR supresses transcription from *acsP1*, whilst activating transcription from *pnrf* (Supporting Information Fig. S3), thus, corroborating our assignment of the RNA polymerase complexes observed in Fig. 4.

### Sequences downstream of position +10 repress expression from the *Salmonella nrf* promoter

The *S. enterica* serovar Typhimurium *nrf* promoter is the least active of the promoters tested and previously we demonstrated that sequences upstream of position −87 do not influence expression as the *Salmonella* promoter lacks the stimulatory upstream IHF III site (Fig. 2) (Browning *et al*., 2006). During the course of this study, we cloned the smaller *pnrf97* promoter fragments (−87 to +10) from *E. coli* K‐12 and EHEC into the low copy number *lac* expression vector pRW224, to generate *lacZ* transcriptional fusions (Fig. 5A). As a control we also generated the *S. enterica* serovar Typhimurium version of this construct, i.e., *pnrf97* STM (Supporting Information Table S1). Constructs were transformed into strains JCB387 and JCB3884 (*narL narP*) and promoter activity was determined in cells grown in minimal medium. Surprisingly, results in Fig. 5B indicated that anaerobic expression from the *pnrf97* STM transcriptional fusion was much higher than the *E. coli* K‐12 derivative, resembling that of *pnrf97* EHEC. This suggests that the *Salmonella* promoter is stronger than previously thought and that expression from the longer *pnrf53* STM fragment might be repressed by an additional factor, which binds downstream of position +10. Note that the introduction of the p14C mutation into the *pnrf97* STM promoter fragment, which disrupts the extended −10 element of the promoter, completely abolished promoter activity, indicating that new promoter elements had not been generated during construction of this fragment (Fig. 5C).

### Expression of the *E. coli* K‐12 and the *Salmonella nrf* operons are regulated by CsrA

**Figure 6:**
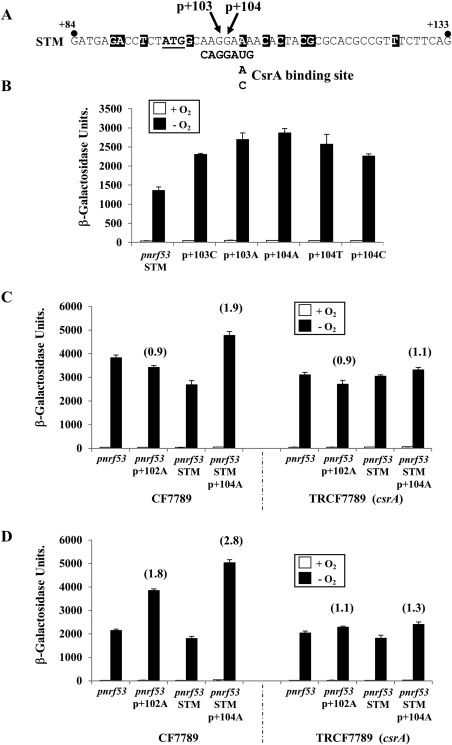
Analysis of CsrA‐dependent regulation of *nrf* operon expression. A. The panel shows the sequence of the *S. enterica* serovar Typhimurium *pnrf53* STM promoter fragment from positions +84 to +133, aligned with the CsrA binding site consensus (Liu *et al*., 1997; Vakulskas *et al*., 2015). The location of the *nrfA* ATG initiation codon is underlined and sequence differences between *pnrf53* STM and the *E. coli* K‐12 fragment are highlighted in black (see also Fig. 2). B. The panel shows the β‐galactosidase activities of JCB3884 (*narL narP*) cells carrying pRW50, containing the *pnrf53* STM promoter fragments harbouring substitutions at positions +103 and +104 (see panel A) cloned as *lacZ* transcriptional fusions. C. The panel shows the β‐galactosidase activities of CF7789 and TRCF7789 (*crsA*) cells, carrying various *pnrf53* and *pnrf53* STM fragments cloned into pRW50 as *lacZ* transcriptional fusions. The p + 102A and p + 104A substitutions disrupt the potential CsrA binding sites in the *pnrf53* and *pnrf53* STM fragments respectively. D. The panel shows the β‐galactosidase activities of CF7789 and TRCF7789 (*crsA*) cells, carrying *pnrf53* and *pnrf53* STM fragments cloned into pRW224 as *lacZ* translational fusions. In all experiments, cells were grown aerobically and anaerobically in minimal salts medium and β‐galactosidase activities are expressed as nmol of ONPG hydrolysed min^−1^ mg^−1^ dry cell mass. Each activity is the average of three independent determinations and standard deviations are shown for all data points. In panels (C) and (D) the fold increase in β‐galactosidase activity, due to the p + 102A or p + 104A substitutions, is indicated in brackets.

CsrA is a sequence‐specific RNA binding protein, which directly represses the translation of many *E. coli* and *Salmonella* genes and can indirectly repress the transcription of others (Lawhon *et al*., 2003; Vakulskas *et al*., 2015). CsrA binds to the consensus sequence CAGGA(U/A/C)G within mRNAs, often found overlapping the ribosome binding sites of the genes it regulates (Liu *et al*., 1997; Vakulskas *et al*., 2015). Inspection of the *S. enterica* serovar Typhimurium *nrfA* sequence, around the ATG translation initiation codon, suggested that it might contain a CsrA binding sequence (Fig. 6A). To investigate this, point mutations, at positions +103 and +104, were introduced into the *pnrf53* STM fragment to disrupt the important GGA motif of the CsrA binding site (Fig. 6A). DNA promoter fragments were cloned into pRW50 to generate *lacZ* transcriptional fusions and β‐galactosidase activity was then examined in JCB3884 (*narL narP*). Results in Fig. 6B show that disruption of the potential CsrA binding site elevated anaerobic expression ∼twofold, suggesting that CsrA might repress expression from the *pnrf53* STM fragment. Note that amino acid sequence of the *S. enterica* serovar Typhimurium CsrA is identical to that of *E. coli* K‐12 (Supporting Information Fig. S2).

To examine whether the *pnrf53* STM fragment is regulated by CsrA, β‐galactosidase expression, from the *pnrf53* STM and the *pnrf53* STMp + 104A fragments (positions −246 to +133), cloned into pRW50, was examined in the Δ*lac* strain CF7789 and in TRCF7789 in which *csrA* is disrupted (Romeo *et al*., 1993). As the sequence surrounding the *E. coli* K‐12 *nrfA* ribosome binding site is similar to that of *Salmonella* (Fig. 2) we also altered the GGA motif in the *E. coli* K‐12 promoter (i.e., the p + 102A substitution). Note that both the p + 104A and p + 102A substitutions maintain the arginine codon at this position in the *nrfA* mRNA when compared to the wild‐type sequence (i.e., AGG verses AGA). Results in Fig. 6C show that expression from *pnrf53* STM p + 104 was elevated ∼twofold in CF7789, whilst no increase was observed in TRCF7789. This suggests that CsrA inhibits expression from the *pnrf53* STM construct. Conversely, the expression from the *E. coli pnrf53* p + 102A fragment was indistinguishable from that of the wild‐type *pnrf53* fragment in the both strains, indicating that CsrA does not regulate expression from the *E. coli* K‐12 *pnrf53* fragment. As CsrA predominantly influences translation (Vakulskas *et al*., 2015), we also cloned each *pnrf53* derivative into pRW224 to generate translational fusions and β‐galactosidase expression was again determined in CF7789 and TRCF7789 (*csrA*). The disruption of the GGA motif in both the *pnrf53* STM and the *E. coli* K‐12 *pnrf53* fragments resulted in an increase of anaerobic expression in CF7789, which was absent in TRCF7789 (Fig. 6D). This indicates that CsrA represses expression from both the *E. coli* and *Salmonella nrf* constructs, when cloned as *lacZ* translational fusions, and suggests that CsrA regulates these two *nrf* operons differently.

To confirm that CsrA regulates both the *E. coli* K‐12 and *Salmonella nrf* operons we examined the effect that over‐expressing CsrA has on expression from the *E. coli* K‐12 *pnrf53* and *pnrf53* STM fragments. A C‐terminal 6His tagged version of *csrA* (*csrA‐6his*) (Dubey *et al*., 2005) was, therefore, cloned into the expression vector pQE60 NdeI to generate pQE60/*csrA* (Raghunathan *et al*., 2011). Induction analysis confirmed that CsrA‐6His could be detected in *E. coli* K‐12 JM109 (*lacI^q^ Δlacz*) cells carrying pQE60/*csrA* (Supporting Information Fig. S4). Therefore, JM109 cells, carrying either pQE60 NdeI or pQE60/*csrA*, were transformed with pRW244 carrying various *pnrf53* and *pnrf53* STM fragments, cloned as translational fusions. Cells were grown anaerobically in minimal salts medium, containing 0.4% glucose, and the effect of leaky uninduced CsrA‐6His expression was determined by measuring β‐galactosidase expression. Results in Table 3 show that CsrA caused a large decrease in expression from the *pnrf53* and *pnrf53* STM wild‐type fragments, when compared to cells carrying the empty pQE60 NdeI vector. However, for *pnrf53* and *pnrf53* STM fragments, carrying substitutions in the CsrA binding site (i.e., *pnrf53* p + 102A and *pnrf53* STM p + 104A respectively) the effect of CsrA expression was considerably less. This confirms that CsrA represses expression of both *E. coli* K‐12 and *Salmonella nrfA* using the CsrA binding sites identified by this study.

**Table 3:** Repression of *pnrf* promoter derivatives by CsrA‐6His expression.

	β‐Galactosidase activity^a^		
Promoter^b^	pQE60 NdeI	pQE60/*csrA*	Ratio^c^
*pnrf53*	516 ± 32	41 ± 3	12.6
*pnrf53* p+102A	1314 ± 90	643 ± 84	2
*pnrf53* STM	399 ± 14	17 ± 1	23.5
*pnrf53* STM p+104A	2269 ± 43	338 ± 1	6.7

**a.** β‐galactosidase activities were measured in strain JM109 (*lacI^q^* Δ*lac*) carrying pRW224 containing different *pnrf53* translational fusions and pQE60 derivatives. Cells were grown anaerobically in minimal salts medium supplemented with 0.4% glucose to control CsrA‐6His expression. β‐galactosidase activities are expressed as nmol of ONPG hydrolysed min^−1^ mg^−1^ dry cell mass, each activity is the average of three independent determinations and standard deviations are shown.

**b.** The first column lists the *pnrf53* fragments used.

**c.** The ratio column indicates the fold repression due to the leaky expression of CsrA‐6His.

### Sequences surrounding the transcription start site of the *Salmonella nrf* promoter are responsible for its elevated promoter activity

**Figure 7:**
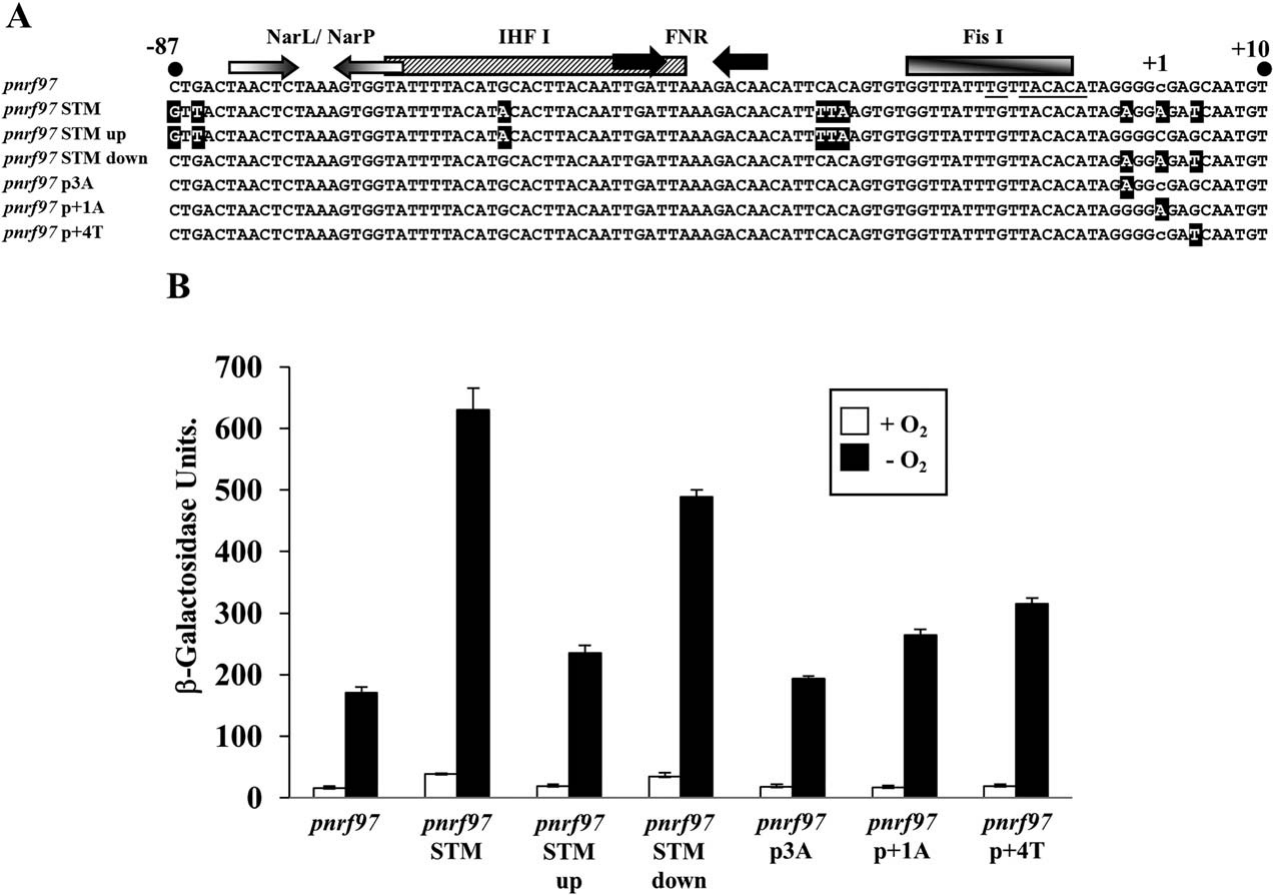
Expression of chimeric *pnrf97* promoter fragments. A. The panel shows the sequence alignment of various wild‐type and chimeric *pnrf97* fragments (positions −87 to +10). The position of the transcription start site for *E. coli* K‐12 *pnrf97* is indicated by lower case text. The location of FNR and NarL/NarP binding sites are represented by inverted arrows, whilst IHF I and Fis I binding sites are depicted by boxes. Differences between the *E. coli pnrf97* fragment and the *pnrf97* STM derivatives are highlighted in black. B. The panel shows the β‐galactosidase activities of JCB3884 (*narL narP*) cells carrying pRW224, containing various *pnrf97* promoter fragments. Cells were grown aerobically and anaerobically in minimal salts medium. β‐galactosidase activities are expressed as nmol of ONPG hydrolysed min^−1^ mg^−1^ dry cell mass, each activity is the average of three independent determinations and standard deviations are indicated.

Results in Fig. 5 indicate that the anaerobic expression from the *Salmonella pnrf97* STM fragment is elevated in comparison to the *E. coli* K‐12 *pnrf97* fragment. To identify which parts of the *Salmonella* promoter that were responsible for this, we generated chimeric *pnrf97* fragments, in which the upstream and downstream sequences from *pnrf97* STM were introduced into the *E. coli* K‐12 *pnrf97* fragment (Fig. 7A). Fragments were cloned into pRW224 to generate *lacZ* transcriptional fusions, and β‐galactosidase activities were determined in JCB3884 (*narL narP*) cells, during aerobic and anaerobic growth in minimal medium. Results in Fig. 7B show that the downstream differences surrounding the transcription start site were predominantly responsible for the increased promoter activity. When individual differences were introduced into the *E. coli* K‐12 *pnrf97* fragment (i.e., the p3A, p + 1A and p + 4T substitutions) only those at positions +1 and +4 led to small increases in expression, in comparison to *pnrf97* (Fig. 7B). Thus, we conclude that the differences around the transcription start site are responsible for the higher promoter activity observed for the *Salmonella pnrf97* fragment and that none of the differences alone are responsible for the elevation observed.

## Discussion

Both the *E. coli* and *Salmonella* formate‐dependent Nrf nitrite reductases can support anaerobic growth on nitrite and detoxify NO, making them important for survival under the anoxic conditions experienced in the intestines of warm blooded animals (Pope and Cole, 1982; Poock *et al*., 2002; Lundberg *et al*., 2004; Gilberthorpe and Poole, 2008; Mills *et al*., 2008; van Wonderen *et al*., 2008). Here, we show that the regulation of the *nrf* promoters from various pathogenic bacteria is similar to that of *E. coli* K‐12, being induced by anaerobiosis and nitrite (Fig. 3). However, in spite of maintaining this general pattern of regulation, the core promoters of the EHEC and *Salmonella* promoters are more active than that of *E. coli* K‐12 (Fig. 5B). Our results also demonstrate that the EHEC *nrf* promoter displays considerable FNR‐independent activity (Table 1), and that the operon is expressed in EHEC in the presence of oxygen (Supporting Information Fig. S1). The EHEC promoter only differs from its *E. coli* K‐12 counterpart due to a single base pair improvement in the −10 element at position −7 (Fig. 2), which is an important base in the −10 hexamer (Shultzaberger *et al*., 2007; Browning and Busby, 2016). This difference increases the affinity of RNA polymerase for the EHEC promoter and enables RNA polymerase to out‐compete Fis when binding, decreasing the effect of this repression (Fig. 4: Table 2). Alignment of promoter sequences from 22 EHEC O157:H7 strains (Supporting Information Fig. S5A) indicated that this improvement was present in all strains examined, however, it was absent from the closely related *E. coli* O55:H7 and EHEC O157:H‐ strains (Supporting Information Fig. S5B) (Rump *et al*., 2011; Sadiq *et al*., 2014). This indicates that this alteration in *pnrf* appears to be specific to EHEC O157:H7. We suggest that, as EHEC O157:H7 is a commensal of cattle, and as cattle feeds often contain elevated nitrate levels, the altered expression patterns of *nrf* may facilitate growth in the presence of nitrite and NO, derived from metabolism in this niche (Lundberg *et al*., 2004; Callaway *et al*., 2009; Cockburn *et al*., 2013).

The core promoter of the *Salmonella nrf* promoter was also much stronger than that of *E. coli* K12, with anaerobic expression rivalling that of the EHEC promoter (Fig. 5B). Chimeric promoter constructs demonstrated that this was primarily due to sequence differences around +1, but that no specific difference was absolutely responsible (Fig. 7B). At bacteria promoters, the occurrence of an A as an initiating nucleotide is favoured over C and this difference could partly explain the strength of the *Salmonella* promoter (Jeong and Kang, 1994; Walker and Osuna, 2002; Vvedenskaya *et al*., 2015). Furthermore, during transcription initiation RNA polymerase also recognizes bases around the transcript start, i.e., the core recognition element, and, thus, differences in this region of the *Salmonella* promoter could influence recognition of this element and in turn affect promoter activity (Zhang *et al*., 2012; Bae *et al*., 2015; Vvedenskaya *et al*., 2016). Alignment of *nrf* promoter sequences from different *S. enterica* serovars (Supporting Information Fig. S6) indicated that many serovars possessed the same differences around +1 as *S. enterica* serovar Typhimurium, indicating that these improvements are largely conserved. However, two *S. enterica* serovars (Dublin and Newport) possess a G at position +4, which is the same as the *E. coli* K‐12 promoter at this position (Supporting Information Fig. S6). Thus, we would expect in these *Salmonella* serovars expression from *pnrf* to be lower.

Our data indicates that although the EHEC and *S. enterica* serovar Typhimurium core promoters have similar anaerobic expression levels (Fig. 5B) they achieve it using different mechanisms, highlighting the ability of bacteria to mix and match their promoter elements to achieve similar transcription outputs (Miroslavova and Busby, 2006; Hook‐Barnard and Hinton, 2007). The EHEC solution to increasing promoter strength, by altering the −10 element, decreases the dependency of the promoter on FNR and its sensitivity to repression during growth in rich media. For the *Salmonella* promoter, changing bases around the transcription start site increases promoter activity but maintains the overall regulation seen at the *E. coli* K‐12 promoter (Tables 1 and 2). Thus, increasing promoter strength to similar levels can result in very different patterns of regulation.

The RNA binding protein, CsrA, regulates the expression of many genes in *E. coli* K‐12 and *S. enterica* serovar Typhimurium, controlling diverse traits such as metabolism, biofilm formation, motility and virulence (Lawhon *et al*., 2003; Edwards *et al*., 2011; Vakulskas *et al*., 2015). Our results demonstrate that CsrA modulates anaerobic respiration in both *E. coli* and *Salmonella* by regulating expression of the formate‐dependent NrfA periplasmic nitrite reductase. Consistent with our results, pull down experiments with 6His tagged CsrA identified *nrfA* mRNA as a target for CsrA in *E. coli* (Edwards *et al*., 2011). The positioning of the CsrA binding site suggests that CsrA likely represses translation, perhaps by preventing ribosomal access to the *nrfA* ribosome binding site in both bacterial species (Figs 2 and 6D). However, it is clear from our work with transcriptional fusions that *Salmonella nrfA* expression is more complicated (Fig. 6C) and that CsrA may influence *nrfA* transcription elongation, perhaps by causing premature transcription termination, as has been observed for *pgaA* in *E. coli*, or alternatively it may affect transcript stability (Figueroa‐Bossi *et al*., 2014; Vakulskas *et al*., 2015). As CsrA exists as a homo‐dimer it is able to associate with two sites separated by up to 63 nucleotides (Mercante *et al*., 2009). It is of note that the *nrfA* untranslated leaders, in both bacteria, contain additional GGA motifs, which could serve as auxiliary low affinity CsrA targets (Fig. 2). This, coupled with any differences in mRNA secondary structure, could account for the apparent differential CsrA regulation observed between the *E. coli* and *Salmonella* constructs.

In *E. coli*, CsrA availability and activity is controlled by the small non‐coding RNAs (sRNA) CsrB and CsrC (CsrB/C), which both contain multiple CsrA binding motifs that sequester CsrA away from its target transcripts (Vakulskas *et al*., 2015). Production of CsrB/C sRNA is increased in response to the accumulation of metabolic carboxylic acids, e.g., formate and acetate (Suzuki *et al*., 2002; Vakulskas *et al*., 2015) and CsrB/C turnover is accelerated by the presence of glucose (Leng *et al*., 2016). Thus, it has been proposed that when a preferred carbon source is exhausted and metabolic carboxylic acids build up, CsrB/C levels increase and sequester CsrA to promote the switch from exponential to stationary phase (Leng *et al*., 2016). As CsrA regulates gene expression in response to nutrient quality, we propose that CsrA has been co‐opted at *nrf* to reinforce operon regulation in response to this signal. For example, during anaerobic growth in rich medium, *nrf* operon transcription is sharply inhibited by Fis (Browning *et al*., 2005). Under these conditions CsrB/C sRNA turnover should be increased and CsrA ‘freed’ to repress its targets, including *nrfA*. However, during growth in poor media *nrf* operon transcription in maximal as Fis levels are lower, particularly in stationary phase (Ball *et al*., 1992; Ali Azam *et al*., 1999). If this is coupled with the accumulation of carboxylic acids, we predict that increased CsrB/C sRNA production and decreased turnover, would sequester CsrA from the *nrf* transcript to allow its increased translation. As NrfA‐mediated nitrite reduction is also dependent on formate, it is possible that CsrA regulation is also a way of linking *nrf* operon expression to formate levels. Thus, we propose the use of CsrA and Fis together at *nrf* ensures that the translational and transcriptional regulation complement and reinforce one another.

It is clear that the regulation of the *E. coli* K‐12 *nrf* operon is complicated, with a total of six global regulators (i.e., FNR, NarL, NarP, IHF, Fis and now CsrA) coordinating expression in response to environmental and metabolic conditions (Browning *et al*., 2002, 2005, 2006). Much of this regulation is conserved between the different pathogenic bacteria studied here. However, it is clear that regulation of the EHEC and *S. enterica* serovar Typhimurium *nrf* operons have been fine‐tuned, presumably in response to the particular niches occupied by each species in the intestines of their host organisms. Although CsrA has been implicated in *nrfA* regulation, it does not account for all the repression observed for the *Salmonella pnrf53* STM construct and at present the role of the long untranslated leader, which is conserved between species, is unclear. Thus, it is likely that additional regulatory mechanisms may operate to control expression of this complex and important operon in enteric bacteria.

## Experimental procedures

### Bacterial strains, growth conditions, plasmids and primers

The bacterial strains, plasmids and promoter fragments used in this work are listed in Supporting Information Table S1 and oligonucleotides are listed in Supporting Information Table S2. Standard methods for cloning and manipulating DNA fragments were used throughout (Sambrook and Russell, 2001). By convention, locations at the *nrf* promoter are labelled with the transcript start point designated as +1, and with upstream and downstream locations prefixed ‘−’ and ‘+’ respectively. Single base substitutions in *pnrf* are denoted pNX, where N is the position of the substitution relative to the transcript start and X is the substituted base in the non‐template strand of the promoter. For routine DNA manipulations and as a source of DNA fragments for gel retardation, fragments were cloned into plasmid pSR (Kolb *et al*., 1995) and for *in vitro* transcriptions, its derivative, pLSR was used (El‐Robh and Busby, 2002). To measure promoter activities, fragments were cloned into the *lac* expression vectors pRW50 and pRW224 (Lodge *et al*., 1992; Islam *et al*., 2011). Derivatives of pSR, pLSR and pQE60 were maintained in host cells using media supplemented with 100 μg ml^−1^ ampicillin, whilst derivatives of pRW50 and pRW224 were maintained with 15 μg ml^−1^ tetracycline. Cells were grown in either minimal medium (minimal salts with 0.4% glycerol, 10% Lennox broth, 40 mM fumarate) (Pope and Cole, 1982) or in Lennox broth (2% (w/v) peptone, (Merck), 1% (w/v) yeast extract (Fisher Scientific) and 170 mM NaCl) supplemented with 0.4% glucose (Squire *et al*., 2009). Where indicated, a final concentration of 2.5 mM sodium nitrite was added to cultures.

### Promoter fragment and plasmid construction

The DNA sequences of the *nrf* promoters from pathogenic enteric bacteria were compiled from xBASE2 (http://xbase.warwick.ac.uk/) (Fig. 1) (Chaudhuri *et al*., 2008). The *nrf* promoter DNA from EHEC, UPEC, EAEC, EPEC, was amplified by PCR using the nrfA Up and nrfA Down primers (Supporting Information Table S2) with genomic DNA as template. The *S. flexneri pnrf* DNA was amplified using nrfSFX Up and nrfA Down primers. PCR products were restricted with EcoRI and BamHI and cloned into pRW50 to generate *lacZ* transcriptional fusions.

The wild‐type *pnrf97* EHEC and *pnrf97* STM fragments were generated by PCR, using the primer pairs nrfA E87/nrfO157 H10 and nrfSTM E87/nrfSTM H10, with the relevant genomic DNA as template. The chimeric *pnrf97* STM up and *pnrf* STM down promoter fragments were also synthesized by PCR, using primer pairs nrfSTM E87/nrfA H10 and nrfA E87/nrfSTM H10 with pRW224/*pnrf97* STM and pRW224/*pnrf97*, respectively, as template. The p3A, p + 1A and p + 4T substitutions were introduced into the *E. coli* K‐12 *pnrf97* fragment by PCR using primers nrfSTM p3A, nrfSTM p + 1A and nrfSTM p + 4T with primer nrfA E87 and pRW224/*pnrf97* as template. The extended −10 motif in the *pnrf97* STM promoter fragment was disrupted by PCR amplifying the *Salmonella* promoter region using primers nrfSTM E87 and nrfSTM p14C with pRW224/*pnrf97* STM as template. All *pnrf97* promoter derivatives were restricted with EcoRI and HindIII and cloned into pRW224 to generate *lacZ* transcriptional fusions.

The *pnrf53* and *pnrf53* STM fragments, carrying substitutions in the CsrA binding site, were generated using megaprimer PCR (Sarkar and Sommer, 1990). In the first round of PCR primers nrfA p + 102A, nrfSTM p + 103, nrfSTM p + 104, were used in conjunction with primer D10527 and the relevant pRW50/*pnrf53* construct. Each PCR product was then used with primer D10520 and the same template to generate the final PCR product, which was restricted with either EcoRI and HindIII for cloning into pRW50 to generate *lacZ* transcriptional fusions or EcoRI and BamHI for pRW224 to generate *lacZ* translational fusions.

To express CsrA *in vivo*, the DNA encoding a C‐terminal 6His tagged version of *csrA* (*csrA‐6his*) was excised from plasmid pCSB12 (Dubey *et al*., 2005) and cloned into vector pQE60 NdeI (Raghunathan *et al*., 2011), using NdeI and BamHI, to generate plasmid pQE60/*csrA*. Plasmid pQE60/*csrA* was maintained in *E. coli* K‐12 JM109 cells, grown in the presence of 0.4 to 1% glucose, to limit the leaky expression of CsrA.

### Assays of *nrf* promoter activity

To assay the expression from *pnrf* derivatives cloned into the *lac* expression vectors pRW50 and pRW224, different host strains were transformed and β‐galactosidase activity was measured as described in our previous work (Jayaraman *et al*., 1987). Cells were grown in either minimal salts medium (Pope and Cole, 1982) or rich medium [Lennox broth supplemented with 0.4% glucose (Squire *et al*., 2009)]. Where indicated, a final concentration of 2.5 mM sodium nitrite was added to cultures. For aerobic growth, cells were shaken vigorously to an OD_650_ of 0.2 to 0.3, whilst, for anaerobic growth, they were held static in growth tubes to an OD_650_ of 0.4 to 0.6. β‐galactosidase activities are reported as nmol of ONPG hydrolysed in our assay conditions min^−1^ mg^−1^ dry cell mass and each activity is the average of three independent determinations.

### Western blotting

To examine the expression of NrfA protein in *E. coli* K‐12 and EHEC strains, bacteria were either grown anaerobically and aerobically in minimal malts medium at 37°C. For anaerobic growth conditions, 50 ml of bacterial culture was grown without shaking to an OD_600_ of 0.5 to 0.6, whilst for aerobic conditions 10 ml cultures were shaken vigorously to an OD_600_ of 0.3 to 0.4. The preparation of normalized total cellular protein samples, their resolution by SDS‐PAGE gels and their Western blotting was carried out as detailed in our previous work (Browning *et al*., 2013). NrfA proteins were detected using anti‐NrfA antiserum raised in rabbit (kindly provided by Jeff Cole) and blots were developed using the ECL Western Blotting Detection System (GE Healthcare).

To examine the expression of CsrA‐6His protein, JM109 cells, carrying pQE60/*csrA* were grown aerobically in 10 ml of Lennox broth supplemented with glucose, where appropriate, until an OD_600_ of ∼0.4. Protein expression was then induced by the addition of IPTG **(**Isopropyl β‐D‐1‐thiogalactopyranoside) to a final concentration of 1 mM for 3 hrs. Total cellular protein samples were prepared and Western blotting was carried out using anti‐6His (C‐terminal) HRP linked antibody (Invitrogen) (Browning *et al*., 2013).

### Purified proteins

*Escherichia coli* RNA polymerase holoenzyme containing σ^70^ was purchased from Epicentre Technologies (Madison, WI) and FNR DA154 and Fis proteins were purified as described previously (Wing *et al*., 2000; Grainger *et al*., 2008). Note that FNR carries the DA154 substitution, which renders FNR active under aerobic conditions (Wing *et al*., 2000).

### Gel retardation assays

Gel retardation assays using purified FNR, Fis and RNA polymerase were carried out as detailed by Browning *et al*. (2008). Purified promoter fragments were end labelled with [γ‐^32^P]‐ATP and approximately 0.5 ng of each fragment was incubated with varying amounts of each protein. The reaction buffer contained l0 mM potassium phosphate (pH 7.5), 100 mM potassium glutamate, 1 mM EDTA, 50 μM DTT, 5% glycerol and 25 μg ml^−1^ herring sperm DNA. The final reaction volume was 10μl. FNR and Fis proteins were incubated at 37°C for 15 minutes, after which RNA polymerase was added and samples were incubated at 37°C for a further 15 minutes. Samples were loaded directly onto a running 6% polyacrylamide gel (12 V cm^−1^), containing 2% glycerol and 0.25 × TBE and analysed using a Bio‐Rad Molecular Imager FX and Quantity One software (Bio‐Rad).

### *In vitro* transcription assays

The *pnrf97* promoter fragment was cloned into plasmid pLSR, such that the divergent *pnrf* and *pascP1* promoters are both cloned upstream of a lambda *oop* transcription terminator (Supporting Information Fig. S3) (El‐Robh and Busby, 2002). Purified FNR D154A protein was then incubated with pLSR/*pnrf97* plasmid (8 nM final concentration) at 37°C for 20 min in a reaction mixture containing 40 mM Tris‐Cl (pH 7.9), 10 mM MgCl_2_, 50 mM KCl, 0.1 mM dithiothreitol (DTT), 0.2 μg of bovine serum albumin (BSA) μl^−1^, 0.5 mM ATP, 0.5 mM CTP, 0.5 mM GTP, 0.05 mM UTP and 5 μCi of [α‐^32^P]UTP, with a final reaction volume of 20 μl. Purified *E. coli* RNAP was then added to a final concentration of 50 nM, and the mixture was incubated at 37°C for a further 20 min, after which it was stopped by the addition of 25 μl of formamide buffer (95% [vol/vol] deionized formamide, 20 mM EDTA, 0.05% [wt/vol] bromophenol blue, 0.05% [wt/vol] xylene cyanol FF). Samples were then loaded onto a 5.5% denaturing polyacrylamide gel, containing 1 × TBE, and were analysed using a Bio‐Rad Molecular Imager FX and Quantity One software (Bio‐Rad).

## Conflict of Interest

The authors declare no conflict of interest.

## Author contributions

DFB and SJWB conceived and designed the research programme. REG, DJL and DFB performed the experiments. DFB and SJWB wrote the manuscript with input from all authors.

## Supporting information

Supporting Information
