# Supplementary material for: Regulation of nrf operon expression in pathogenic enteric bacteria: sequence divergence reveals new regulatory complexity
Source: Mol Microbiol. 2017 Mar 1;104(4):580–94. doi: 10.1111/mmi.13647 (PMC5434802; doi:10.1111/mmi.13647)
Supplement: Supplementary file 1 — Supporting Information [file MMI-104-580-s001.pdf]

For Molecular Microbiology

Regulation of *nrf* operon expression in pathogenic enteric bacteria: sequence divergence reveals new regulatory complexity.

Supplementary Information

Rita E. Godfrey[1], David J. Lee[1,2], Stephen J. W. Busby[1] and Douglas F. Browning[1]*

[1] Institute of Microbiology and Infection, School of Biosciences, University of Birmingham, Birmingham, B15 2TT, UK.

[2] Department of Life Sciences, School of Health Sciences, Birmingham City University, Birmingham, B15 3TN, UK.

* For correspondence: Tel: +44 (0)121-414-5435.

Email: D.F.Browning@bham.ac.uk

Table S1. Strains, plasmids and promoter fragments used in this work.

Bacterial strains.	Relevant genotype or description.	Reference or source.
JCB387	*E. coli* K-12 Δ*nir* Δ*lac*	(Page *et al.*, 1990)
JCB387Δ*fnr*	JCB387 Δ*fnr*	(Squire *et al.*, 2009)
JCB3884	JCB387 *narL narP253*::Tn*10*d(Cm)	(Tyson *et al.*, 1994)
JM109	*E. coli* K-12 F´ *traD36 proA*$^{+}$*B*$^{+}$ *lacI*^{q} *Δ(lacZ)M15/ Δ(lac-proAB) glnV44 e14*$^{-}$ *gyrA96 recA1 relA1 endA1 thi hsdR17*	New England Biolabs.
JRG1728	*E. coli* K-12 Δ*fnr*	(Wing *et al.*, 1995)
RK4353	*E. coli* K-12 *ΔlacUl69 araDJ39 rpsL gyrA non*	(Stewart and MacGregor, 1982)
JCB5205	RK4353 *ΔnrfAB*	(Vine *et al.*, 2011)
MG1655	*E. coli* K-12 F^{-} λ^{-} *ilvG*$^{-}$ *rfb-50 rph-1*	(Blattner *et al.*, 1997)
CF7789	MG1655 Δ*lacI-Z* [MluI]	Paul Babitzke.
TRCF7789	CF7789 *csrA*::*kan*	(Romeo *et al.*, 1993)
EHEC EDL933	*E. coli* strain O157:H7 EDL933	(Perna *et al.*, 2001)
EHEC Sakai	*E. coli* strain O157:H7 Sakai	Jon Hobman.
UPEC	*E. coli* strain CFT073	Ian Henderson.
EAEC	*E. coli* strain 042	Ian Henderson.
EPEC	*E. coli* strain E2348/69	Ian Henderson.
S. *flexneri*	S. *flexneri* strain strain 2457T	Ian Henderson.
S. enterica	*S. enterica* serovar Typhimurium strain LT2	Ian Henderson.
**Bacterial plasmids.**		
pSR	pBR322 derivative containing a λ *oop* transcription terminator.	(Kolb *et al.*, 1995)
pLST	pSR derivative containing two λ *oop* transcription terminators.	(El-Robh and Busby, 2002)
pRW50	Broad-host-range *lacZ* transcription fusion vector.	(Lodge *et al.*, 1992)
pRW224	Broad-host-range *lacZ* transcription/ translation fusion vector.	(Islam *et al.*, 2011)
pCSB12	pET21a carrying a C-terminal 6His tagged version of *csrA* (*csrA-6his*).	(Dubey *et al.*, 2005)

pQE60 NdeI	pQE60 with an added NdeI restriction site in its multiple-cloning site.	(Raghunathan *et al.*, 2011)
pQE60/*csrA*	pQE60 NdeI carrying *csrA-6his*	This study
**Promoters fragments.**		
*pnrf*53	*E. coli* K-12 *nrfA* promoter fragment carrying nucleotide sequences from –209 to +131.	(Tyson *et al.*, 1994)
*pnrf*53 EHEC	*E. coli* strain O157:H7 *nrfA* promoter fragment carrying nucleotide sequences from –209 to +131.	This work.
*pnrf*53 UPEC	*E. coli* strain CFT073 *nrfA* promoter fragment carrying nucleotide sequences from –209 to +130.	This work.
*pnrf*53 EAEC	*E. coli* strain 042 *nrfA* promoter fragment carrying nucleotide sequences from –209 to +130.	This work.
*pnrf*53 EPEC	*E. coli* strain E2348/69 *nrfA* promoter fragment carrying nucleotide sequences from –209 to +130.	This work.
*pnrf*53 SFX	S. *flexneri nrfA* promoter fragment carrying nucleotide sequences from –209 to +131.	This work.
pnrf53 STM	*S. enterica* serovar Typhimurium *nrfA* promoter fragment carrying nucleotide sequences from -246 to +133.	(Browning *et al.*, 2006)
pnrf97	*E. coli* K-12 *nrfA* promoter fragment carrying nucleotide sequences from –87 to +10.	(Browning *et al.*, 2002)
pnrf97 p14C	*E. coli* K-12 *nrfA* promoter fragment carrying nucleotide sequences from –87 to +10 and the p14C substitution that disrupts the *pnrf* -10 element.	(Browning *et al.*, 2002)
pnrf97 p63C	*E. coli* K-12 *nrfA* promoter fragment carrying nucleotide sequences from –87 to +10 and the p63 substitution that disrupts the *acsP1*-10 element.	(Browning *et al.*, 2002)
pnrf97 EHEC	*E. coli* strain O157:H7 *nrfA* promoter fragment carrying nucleotide sequences from –87 to +10.	This work.
pnrf97 STM	*S. enterica* serovar Typhimurium *nrfA* promoter fragment carrying nucleotide sequences from -87 to +10.	This work.

pnrf97 STM up	A chimeric *pnrf97* promoter fragment carrying *S. enterica* serovar Typhimurium *nrfA* sequences from -87 to -4 and *E. coli* K-12 sequences from -3 to +10.	This work.
pnrf97 STM down	A chimeric *pnrf97* promoter fragment carrying *E. coli* K-12 *nrfA* sequences from -87 to -4 and *S. enterica* serovar Typhimurium sequences from -3 to +10.	This work.

Table S2. DNA Primers used in this work. Primers (all are shown 5' to 3').

**Primer name.**	**Sequence [a,b].**
D10520	CCCTGCGGTGCCCCTCAAG
D10527	GCAGGTCGTTGAACTGAGCCTGAAATTCAG
nrfA Up	CCCGAATTCCCGGGGATCACGCAAAAGTAGAATTGTGC
nrfA Down	CCCGGATCCCTGAAGATACGGCGTGCG
nrfA E87	CCCGAATTCCTGACTAACTCTAAAGTGG
nrfA H10	CCCAAGCTTACATTGCTCGCCCCTATGTG
nrfA p+102A	CGGATGAAGCCCCTATGACAAGAATAAAAATAAACGCACGCCG
nrfO157 H10	CCCAAGCTTACATTGCTCGCCCCTAAGTGTAACAAA
nrfSFX Up	CCCGAATTCCCGGGGATCACGCAAAAGTAGAATGGTGC
nrfSTM E87	CCCGAATTCGTTACTAACTCTAAAGTGG
nrfSTM H10	CCCAAGCTTACATTGATCTCCTCTATGTG
nrfSTM p14C	GGGGGAAGCTTACATTGATCTCCTCTATGTGTAAGAAATAACC
nrfSTM p+1A	CCCAAGCTTACATTGCTCTCCCCTATGTG
nrfSTM p+4T	CCCAAGCTTACATTGATCGCCCCTATGTG
nrfSTM p3A	CCCAAGCTTACATTGCTCGCCTCTATGTG
nrfSTM p+103	CTGGATGAGACCTCTATGGCAAHGAAAACACTACGCG
nrfSTM p+104	CTGGATGAGACCTCTATGGCAAGHAAAACACTACGCG

[a] Restriction sites are underlined

[b] H=A, C or T.

Supplementary Figure legends

Fig. S1. Detection of NrfA in different *E. coli* strains. The figure shows Western blots and SDS-PAGE gels of normalised total cell protein from *E. coli* K-12 strains (RK4353, JCB5205 (Δ*nrfAB*) and MG1655) and EHEC strain EDL933, grown under (A) anaerobic and (B) aerobic growth conditions. Upper panels show Western blots that were probed with anti-NrfA antiserum to detect full length unprocessed NrfA (NrfA) and the processed mature NrfA (NrfA-pro). Note that due to the higher expression of NrfA under anaerobic conditions 10-fold less sample has been loaded in the Western blot in panel (A) than that in panel (B). In each panel a Coomassie blue SDS-PAGE gel of samples has been included to show that comparable protein levels were obtained for each sample. Total cellular protein samples were prepared and Western blotting was carried out as detailed in our previous work (Browning *et al.*, 2013).

Fig. S2. Comparison of the FNR, Fis and CsrA proteins and RNA polymerase subunits from *E. coli* K-12 strain MG1655, EHEC O157:H7 and *S. enterica* serovar Typhimurium strain LT2. The figure shows an alignment of the protein sequences of (A) FNR, (B) Fis, (C) CsrA, (D) the RNA polymerase α subunit (RpoA), (E) the β subunit (RpoB), (F) the β' subunit (RpoC), (G) the σ^{70} subunit (RpoD) and (H) the ω subunit (RpoZ) from *E. coli* K-12 strain MG1655 (K-12), EHEC O157:H7 (EHEC) and *S. enterica* serovar Typhimurium strain LT2 (ST).

Fig. S3. *In vitro* transcription analysis of the *E. coli* K-12 *nrf-acs* intergenic region. (A) The panel shows the organisation of the pLSR/ *pnrf97* plasmid used for *in vitro* transcription analysis. The *E. coli* K-12 *pnrf97* promoter fragment was cloned into pLSR such that the divergent *pnrf* and *acsP1* promoters drive transcription into pLSR and terminate at the two λ *oop* terminator sequences within the plasmid. Note RNA polymerase is able to transcribe the RNA I transcript on the plasmid, which serves as an internal control for each reaction. (B) The figure shows a multi-round *in vitro* transcription assay using purified FNR DA154 and RNA polymerase with pLSR/ *nrf97* as template. Reactions contained increasing concentrations of FNR protein: lane 1, no protein; lane 2, 0.25 µM; lane 3, 0.5 µM; lane 4, 1 µM; lanes 5, 2 µM. The concentration of RNA polymerase in each reaction was 50 nM. The experiment shows that increasing concentrations of FNR stimulates transcription from *pnrf* but leads to the repression of *acsP1*. (C) The figure shows a multi-round *in vitro* transcription assay using purified FNR DA154 and RNA polymerase with various pLSR/ *nrf97* plasmids as template: lanes 1-3, pLSR/ *pnrf97* p14C; lanes, 4-6, pLSR/ *pnrf97*; lanes 7-9, pLSR/ *pnrf97* p63C. The p14C and p63C substitutions disrupt

the *pnrf* and *acsP1* -10 promoter elements, respectively (Browning *et al.*, 2002). Reactions contained increasing concentrations of FNR protein: lanes 1, 4 and 7, no protein; lanes 2, 5 and 8, 0.25 µM; lanes 3, 6 and 9, 0.5 µM. The concentration of RNA polymerase in each reaction was 50 nM. The experiment shows that disruption of the *pnrf* and *acsP1* -10 promoter element prevents transcription initiation and confirms that the detected transcripts originate from these two promoters.

Fig. S4. Induction analysis of CsrA-6His expression in JM109 pQE60/ *csrA* cells. The figure show the expression of CsrA-6His protein in JM109 cells carrying pQE60/ *csrA* as analysed using a Coomassie blue stained 18% SDS-PAGE gel (upper panel) and Western blotting with anti-6His (C-terminal) HRP antibody (bottom panel). Cells were grown in 10 ml of Lennox broth, supplemented with various glucose concentrations to control CsrA-6His expression (as shown), and protein induction was induced by the addition of 1 mM IPTG (final concentration), where applicable. Total cellular protein samples were prepared and Western blotting was carried out as detailed in our previous work (Browning *et al.*, 2013). Samples were loaded as follows: lanes 2 and 3, JM109 pQE60 NdeI cells (empty vector); lanes 4 to 9, JM109 pQE60/ *csrA* cells.

Fig. S5. Alignment of *nrf* promoter sequences from different EHEC strains. (A) The panel shows the sequence of the *E. coli* K-12 strain MG1655 *nrf* core promoter from positions -57 to +3, aligned with the corresponding *nrf* promoter regions from various EHEC O157:H7 strains. (B) The panel shows the sequence of the *E. coli* K-12 strain MG1655 *nrf* core promoter from positions -57 to +3, aligned with the corresponding *nrf* promoter regions from *E. coli* O55:H7 strains CB9615, 3256-97 and USDA5905, EHEC O157:H- strains 493/89 and H2687, and the EHEC O157:H7 strains G5101, Sakai, EDL933, TW14359 and EC4115. In both panels the location of the FNR binding site, the extended -10 element and the transcription start site is indicated. The differences between the MG1655 *nrf* promoter sequences and those of EHEC are highlighted in red. DNA sequences were obtained from (http://xbase.warwick.ac.uk/) (Chaudhuri *et al.*, 2008) and EcoCyc (https://ecocyc.org/) (Keseler *et al.*, 2013).

Fig. S6. Alignment of the *nrf* promoter sequences from different *S. enterica* serovars. The figure shows the sequence of the *S. enterica* serovar Typhimurium *nrf* core promoter from positions -54 to +6, aligned with the corresponding *nrf* promoter regions from various *S. enterica* serovars and *E. coli* K-12 strain MG1655. The location of the FNR binding site, the extended -10 element and the transcription start site is indicated. The differences between the *S. enterica* serovar Typhimurium promoter and the

other *S. enterica* and *E. coli* K-12 promoter sequences are highlighted in red. DNA sequences were obtained from (http://xbase.warwick.ac.uk/) (Chaudhuri *et al.*, 2008) and EcoCyc (https://ecocyc.org/) (Keseler *et al.*, 2013). The strains used were as follows: *S. enterica* serovar Typhimurium strain LT2, *S. enterica* serovar Heidelberg strain SL476, *S. enterica* serovar Gallinarum strain 287/91, *S. enterica* serovar Enteritidis strain P125109, *S. enterica* serovar Choleraesuis strain SC-B67, *S. enterica* serovar Typhi strain CT18, *S. enterica* serovar Paratyphi A strain AKU 12601, *S. enterica* serovar Paratyphi B strain SPB7, *S. enterica* serovar Paratyphi C strain RKS4594, *S. enterica* serovar Schwarzengrund strain CVM19633, *S. enterica* serovar Dublin strain CT02021853, *S. enterica* serovar Newport strain SL254 and *E. coli* K-12 strain MG1655.

Supplementary References.

Blattner, F.R., Plunkett, G., 3rd, Bloch, C.A., Perna, N.T., Burland, V., Riley, M., Collado-Vides, J., Glasner, J.D., Rode, C.K., Mayhew, G.F., Gregor, J., Davis, N.W., Kirkpatrick, H.A., Goeden, M.A., Rose, D.J., Mau, B. and Shao, Y. (1997) The complete genome sequence of *Escherichia coli* K-12. *Science* **277**: 1453-1462.

Browning, D.F., Beatty, C.M., Wolfe, A.J., Cole, J.A. and Busby, S.J. (2002) Independent regulation of the divergent *Escherichia coli nrfA* and *acsP1* promoters by a nucleoprotein assembly at a shared regulatory region. *Mol Microbiol* **43**: 687-701.

Browning, D.F., Lee, D.J., Wolfe, A.J., Cole, J.A. and Busby, S.J. (2006) The *Escherichia coli* K-12 NarL and NarP proteins insulate the *nrf* promoter from the effects of integration host factor. *J Bacteriol* **188**: 7449-7456.

Browning, D.F., Matthews, S.A., Rossiter, A.E., Sevastsyanovich, Y.R., Jeeves, M., Mason, J.L., Wells, T.J., Wardius, C.A., Knowles, T.J., Cunningham, A.F., Bavro, V.N., Overduin, M. and Henderson, I.R. (2013) Mutational and topological analysis of the *Escherichia coli* BamA protein. *PLoS One* **8**: e84512.

Chaudhuri, R.R., Loman, N.J., Snyder, L.A., Bailey, C.M., Stekel, D.J. and Pallen, M.J. (2008) xBASE2: a comprehensive resource for comparative bacterial genomics. *Nucleic Acids Res* **36**: D543-546.

Dubey, A.K., Baker, C.S., Romeo, T. and Babitzke, P. (2005) RNA sequence and secondary structure participate in high-affinity CsrA-RNA interaction. *Rna* **11**: 1579-1587.

El-Robh, M.S. and Busby, S.J. (2002) The *Escherichia coli* cAMP receptor protein bound at a single target can activate transcription initiation at divergent promoters: a systematic study that exploits new promoter probe plasmids. *Biochem J* **368**: 835-843.

Islam, M.S., Bingle, L.E., Pallen, M.J. and Busby, S.J. (2011) Organization of the LEE1 operon regulatory region of enterohaemorrhagic *Escherichia coli* O157:H7 and activation by GrlA. *Mol Microbiol* **79**: 468-483.

Keseler, I.M., Mackie, A., Peralta-Gil, M., Santos-Zavaleta, A., Gama-Castro, S., Bonavides-Martinez, C., Fulcher, C., Huerta, A.M., Kothari, A., Krummenacker, M., Latendresse, M., Muniz-Rascado, L., Ong, Q., Paley, S., Schroder, I., Shearer, A.G., Subhraveti, P., Travers, M., Weerasinghe, D., Weiss, V., Collado-Vides, J., Gunsalus, R.P., Paulsen, I. and Karp, P.D. (2013) EcoCyc: fusing model organism databases with systems biology. *Nucleic Acids Res* **41**: D605-612.

Kolb, A., Kotlarz, D., Kusano, S. and Ishihama, A. (1995) Selectivity of the *Escherichia coli* RNA polymerase E sigma 38 for overlapping promoters and ability to support CRP activation. *Nucleic Acids Res* **23**: 819-826.

Lodge, J., Fear, J., Busby, S., Gunasekaran, P. and Kamini, N.R. (1992) Broad host range plasmids carrying the *Escherichia coli* lactose and galactose operons. *FEMS Microbiol Lett* **74**: 271-276.

Page, L., Griffiths, L. and Cole, J.A. (1990) Different physiological roles of two independent pathways for nitrite reduction to ammonia by enteric bacteria. *Arch Microbiol* **154**: 349-354.

Perna, N.T., Plunkett, G., 3rd, Burland, V., Mau, B., Glasner, J.D., Rose, D.J., Mayhew, G.F., Evans, P.S., Gregor, J., Kirkpatrick, H.A., Posfai, G., Hackett, J., Klink, S., Boutin, A., Shao, Y., Miller, L., Grotbeck, E.J., Davis, N.W., Lim, A., Dimalanta, E.T., Potamousis,

K.D., Apodaca, J., Anantharaman, T.S., Lin, J., Yen, G., Schwartz, D.C., Welch, R.A. and Blattner, F.R. (2001) Genome sequence of enterohaemorrhagic *Escherichia coli* O157:H7. *Nature* **409**: 529-533.

Raghunathan, D., Wells, T.J., Morris, F.C., Shaw, R.K., Bobat, S., Peters, S.E., Paterson, G.K., Jensen, K.T., Leyton, D.L., Blair, J.M., Browning, D.F., Pravin, J., Flores-Langarica, A., Hitchcock, J.R., Moraes, C.T., Piazza, R.M., Maskell, D.J., Webber, M.A., May, R.C., MacLennan, C.A., Piddock, L.J., Cunningham, A.F. and Henderson, I.R. (2011) SadA, a trimeric autotransporter from *Salmonella enterica* serovar Typhimurium, can promote biofilm formation and provides limited protection against infection. *Infect Immun* **79**: 4342-4352.

Romeo, T., Gong, M., Liu, M.Y. and Brun-Zinkernagel, A.M. (1993) Identification and molecular characterization of *csrA*, a pleiotropic gene from *Escherichia coli* that affects glycogen biosynthesis, gluconeogenesis, cell size, and surface properties. *J Bacteriol* **175**: 4744-4755.

Squire, D.J., Xu, M., Cole, J.A., Busby, S.J. and Browning, D.F. (2009) Competition between NarL-dependent activation and Fis-dependent repression controls expression from the *Escherichia coli yeaR* and *ogt* promoters. *Biochem J* **420**: 249-257.

Stewart, V. and MacGregor, C.H. (1982) Nitrate reductase in *Escherichia coli* K-12: involvement of *chlC*, *chlE*, and *chlG* loci. *J Bacteriol* **151**: 788-799.

Tyson, K.L., Cole, J.A. and Busby, S.J. (1994) Nitrite and nitrate regulation at the promoters of two *Escherichia coli* operons encoding nitrite reductase: identification of common target heptamers for both NarP- and NarL-dependent regulation. *Mol Microbiol* **13**: 1045-1055.

Vine, C.E., Purewal, S.K. and Cole, J.A. (2011) NsrR-dependent method for detecting nitric oxide accumulation in the *Escherichia coli* cytoplasm and enzymes involved in NO production. *FEMS Microbiol Lett* **325**: 108-114.

Wing, H.J., Williams, S.M. and Busby, S.J. (1995) Spacing requirements for transcription activation by *Escherichia coli* FNR protein. *J Bacteriol* **177**: 6704-6710.

Figure S1.

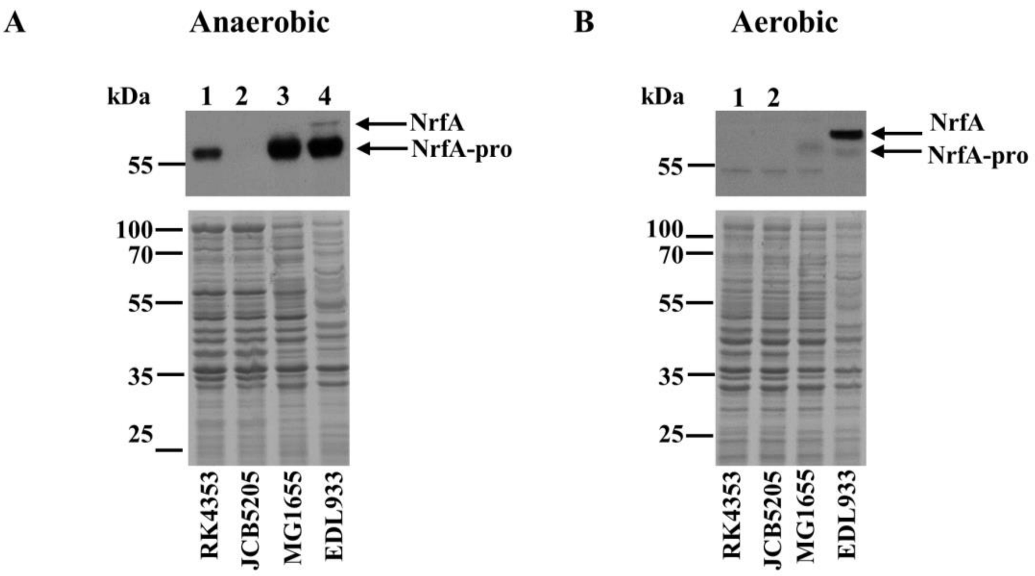
A
Anaerobic
kDa
1
2
3
4
NrfA
NrfA-pro
55
100
70
55
35
25
RK4353
JCB5205
MG1655
EDL933
B
Aerobic
kDa
1
2
NrfA
NrfA-pro
55
100
70
55
35
25
RK4353
JCB5205
MG1655
EDL933

Figure S2.

A FNR

```
FNR K-12        MIPEKRIIRRIQSGGCAIHCQDCSISQLCIPFTLNEHELDQLDNIIERKKPIQKGQTLFK
FNR EHEC        MIPEKRIIRRIQSGGCAIHCQDCSISQLCIPFTLNEHELDQLDNIIERKKPIQKGQTLFK
FNR ST          MIPEKRIIRRIQSGGCAIHCQDCSISQLCIPFTLNEHELDQLDNIIERKKPIQKGQTLFK
                ************************************************************

FNR K-12        AGDELKSLYAIRSGTIKSYTITEQGDEQITGFHLAGDLVGFDAIGSGHHPSFAQALETSM
FNR EHEC        AGDELKSLYAIRSGTIKSYTITEQGDEQITGFHLAGDLVGFDAIGSGHHPSFAQALETSM
FNR ST          AGDELKSLYAIRSGTIKSYTITEQGDEQITGFHLAGDLVGFDAIGSGHHPSFAQALETSM
                ************************************************************

FNR K-12        VCEIPFETLDDLSGKMPNLRQQMMRLMSGEIKGDQDMILLLSKKNAEERLAAFIYNLSRR
FNR EHEC        VCEIPFETLDDLSGKMPNLRQQMMRLMSGEIKGDQDMILLLSKKNAEERLAAFIYNLSRR
FNR ST          VCEIPFETLDDLSGKMPNLRQQMMRLMSGEIKGDQDMILLLSKKNAEERLAAFIYNLSRR
                ************************************************************

FNR K-12        FAQRGFSPREFRLTMTRGDIGNYLGLTVETISRLLGRFQKSGMLAVKGKYITIENNDALA
FNR EHEC        FAQRGFSPREFRLTMTRGDIGNYLGLTVETISRLLGRFQKSGMLAVKGKYITIENNDALA
FNR ST          FAQRGFSPREFRLTMTRGDIGNYLGLTVETISRLLGRFQKSGMLAVKGKYITIENSDALA
                *******************************************************.****

FNR K-12        QLAGHTRNVA
FNR EHEC        QLAGHTRNVA
FNR ST          ALAGHTRNVA
                 *********
```

B Fis

```
Fis K-12        MFEQRVNSDVLTVSTVNSQDQVTQKPLRDSVKQALKNYFAQLNGQDVNDLYELVLAEVEQ
Fis ST          MFEQRVNSDVLTVSTVNSQDQVTQKPLRDSVKQALKNYFAQLNGQDVNDLYELVLAEVEQ
Fis EHEC        MFEQRVNSDVLTVSTVNSQDQVTQKPLRDSVKQALKNYFAQLNGQDVNDLYELVLAEVEQ
                ************************************************************

Fis K-12        PLLDMVMQYTRGNQTRAALMMGINRGTLRKKLKKYGMN
Fis ST          PLLDMVMQYTRGNQTRAALMMGINRGTLRKKLKKYGMN
Fis EHEC        PLLDMVMQYTRGNQTRAALMMGINRGTLRKKLKKYGMN
                **************************************
```

C CsrA

```
CsrA K-12       MLILTRRVGETLMIGDEVTVTVLGVKGNQVRIGVNAPKEVSVHREEIYQRIQAEKSQQSS
CsrA ST         MLILTRRVGETLMIGDEVTVTVLGVKGNQVRIGVNAPKEVSVHREEIYQRIQAEKSQQSS
CsrA EHEC       MLILTRRVGETLMIGDEVTVTVLGVKGNQVRIGVNAPKEVSVHREEIYQRIQAEKSQQSS
                ************************************************************

CsrA K-12       Y
CsrA ST         Y
CsrA EHEC       Y
                *
```

D **RNA polymerase α subunit/ RpoA**

```
RpoA K-12       MQGSVTEFLKPRLVDIEQVSSTHAKVTLEPLERGFGHTLGNALRRILLSSMPGCAVTEVE
RpoA ST         MQGSVTEFLKPRLVDIEQVSSTHAKVTLEPLERGFGHTLGNALRRILLSSMPGCAVTEVE
RpoA EHEC       MQGSVTEFLKPRLVDIEQVSSTHAKVTLEPLERGFGHTLGNALRRILLSSMPGCAVTEVE
                ************************************************************

RpoA K-12       IDGVLHEYSTKEGVQEDILEILLNLKGLAVRVQGKDEVILTLNKSGIGPVTAADITHDGD
RpoA ST         IDGVLHEYSTKEGVQEDILEILLNLKGLAVRVQGKDEVILTLNKSGIGPVTAADITHDGD
RpoA EHEC       IDGVLHEYSTKEGVQEDILEILLNLKGLAVRVQGKDEVILTLNKSGIGPVTAADITHDGD
                ************************************************************

RpoA K-12       VEIVKPQHVICHLTDENASISMRIKVQRGRGYVPASTRIHSEEDERPIGRLLVDACYSPV
RpoA ST         VEIVKPQHVICHLTDENASISMRIKVQRGRGYVPASTRIHSEEDERPIGRLLVDACYSPV
RpoA EHEC       VEIVKPQHVICHLTDENASISMRIKVQRGRGYVPASTRIHSEEDERPIGRLLVDACYSPV
                ************************************************************

RpoA K-12       ERIAYNVEAARVEQRTDLDKLVIEMETNGTIDPEEAIRRAATILAEQLEAFVDLRDVRQP
RpoA ST         ERIAYNVEAARVEQRTDLDKLVIEMETNGTIDPEEAIRRAATILAEQLEAFVDLRDVRQP
RpoA EHEC       ERIAYNVEAARVEQRTDLDKLVIEMETNGTIDPEEAIRRAATILAEQLEAFVDLRDVRQP
                ************************************************************

RpoA K-12       EVKEEKPEFDPILLRPVDDLELTVRSANCLKAEAIHYIGDLVQRTEVELLKTPNLGKKSL
RpoA ST         EVKEEKPEFDPILLRPVDDLELTVRSANCLKAEAIHYIGDLVQRTEVELLKTPNLGKKSL
RpoA EHEC       EVKEEKPEFDPILLRPVDDLELTVRSANCLKAEAIHYIGDLVQRTEVELLKTPNLGKKSL
                ************************************************************

RpoA K-12       TEIKDVLASRGLSLGMRLENWPPASIADE
RpoA ST         TEIKDVLASRGLSLGMRLENWPPASIADE
RpoA EHEC       TEIKDVLASRGLSLGMRLENWPPASIADE
                *****************************
```

E **RNA polymerase β subunit / RpoB**

```
RpoB K-12       MVYSYTEKKRIRKDFGKRPQVLDVPYLLSIQLDSFQKFIEQDPEGQYGLEAAFRSVFPIQ
RpoB EHEC       MVYSYTEKKRIRKDFGKRPQVLDVPYLLSIQLDSFQKFIEQDPEGQYGLEAAFRSVFPIQ
RpoB ST         MVYSYTEKKRIRKDFGKRPQVLDVPYLLSIQLDSFQKFIEQDPEGQYGLEAAFRSVFPIQ
                ************************************************************

RpoB K-12       SYSGNSELQYVSYRLGEPVFDVQECQIRGVTYSAPLRVKLRLVIYEREAPEGTVKDIKEQ
RpoB EHEC       SYSGNSELQYVSYRLGEPVFDVQECQIRGVTYSAPLRVKLRLVIYEREAPEGTVKDIKEQ
RpoB ST         SYSGNSELQYVSYRLGEPVFDVQECQIRGVTYSAPLRVKLRLVIYEREAPEGTVKDIKEQ
                ************************************************************

RpoB K-12       EVYMGEIPLMTDNGTFVINGTERVIVSQLHRSPGVFFDSDKGKTHSSGKVLYNARIIPYR
RpoB EHEC       EVYMGEIPLMTDNGTFVINGTERVIVSQLHRSPGVFFDSDKGKTHSSGKVLYNARIIPYR
RpoB ST         EVYMGEIPLMTDNGTFVINGTERVIVSQLHRSPGVFFDSDKGKTHSSGKVLYNARIIPYR
                ************************************************************

RpoB K-12       GSWLDFEFDPKDNLFVRIDRRRKLPATIILRALNYTTEQILDLFFEKVIFEIRDNKLQME
RpoB EHEC       GSWLDFEFDPKDNLFVRIDRRRKLPATIILRALNYTTEQILDLFFEKVIFEIRDNKLQME
RpoB ST         GSWLDFEFDPKDNLFVRIDRRRKLPATIILRALNYTTEQILDLFFEKVVFEIRDNKLQME
                ************************************************:***********

RpoB K-12       LVPERLRGETASFDIEANGKVYVEKGRRITARHIRQLEKDDVKLIEVPVEYIAGKVVAKD
RpoB EHEC       LVPERLRGETASFDIEANGKVYVEKGRRITARHIRQLEKDDVKLIEVPVEYIAGKVVAKD
RpoB ST         LIPERLRGETASFDIEANGKVYVEKGRRITARHIRQLEKDDIKHIEVPVEYIAGKVVSKD
                *:***************************************:* *************:**

RpoB K-12       YIDESTGELICAANMELSLDLLAKLSQSGHKRIETLFTNDLDHGPYISETLRVDPTNDRL
RpoB EHEC       YIDESTGELICAANMELSLDLLAKLSQSGHKRIETLFTNDLDHGPYISETLRVDPTNDRL
RpoB ST         YVDESTGELICAANMELSLDLLAKLSQSGHKRIETLFTNDLDHGPYISETVRVDPTNDRL
                *:************************************************:*********
```

```
RpoB K-12       SALVEIYRMMRPGEPPTREAAESLFENLFFSEDRYDLSAVGRMKFNRSLLREEIEGSGIL
RpoB EHEC       SALVEIYRMMRPGEPPTREAAESLFENLFFSEDRYDLSAVGRMKFNRSLLREEIEGSGIL
RpoB ST         SALVEIYRMMRPGEPPTREAAESLFENLFFSEDRYDLSAVGRMKFNRSLLRDEIEGSGIL
                ***************************************************:********

RpoB K-12       SKDDIIDVMKKLIDIRNGKGEVDDIDHLGNRRIRSVGEMAENQFRVGLVRVERAVKERLS
RpoB EHEC       SKDDIIDVMKKLIDIRNGKGEVDDIDHLGNRRIRSVGEMAENQFRVGLVRVERAVKERLS
RpoB ST         SKDDIIDVMKKLIDIRNGKGEVDDIDHLGNRRIRSVGEMAENQFRVGLVRVERAVKERLS
                ************************************************************

RpoB K-12       LGDLDTLMPQDMINAKPISAAVKEFFGSSQLSQFMDQNNPLSEITHKRRISALGPGGLTR
RpoB EHEC       LGDLDTLMPQDMINAKPISAAVKEFFGSSQLSQFMDQNNPLSEITHKRRISALGPGGLTR
RpoB ST         LGDLDTLMPQDMINAKPISAAVKEFFGSSQLSQFMDQNNPLSEITHKRRISALGPGGLTR
                ************************************************************

RpoB K-12       ERAGFEVRDVHPTHYGRVCPIETPEGPNIGLINSLSVYAQTNEYGFLETPYRKVTDGVVT
RpoB EHEC       ERAGFEVRDVHPTHYGRVCPIETPEGPNIGLINSLSVYAQTNEYGFLETPYRKVTDGVVT
RpoB ST         ERAGFEVRDVHPTHYGRVCPIETPEGPNIGLINSLSVYAQTNEYGFLETPYRRVVDGVVT
                ****************************************************:*.*****

RpoB K-12       DEIHYLSAIEEGNYVIAQANSNLDEEGHFVEDLVTCRSKGESSLFSRDQVDYMDVSTQQV
RpoB EHEC       DEIHYLSAIEEGNYVIAQANSNLDEEGHFVEDLVTCRSKGESSLFSRDQVDYMDVSTQQV
RpoB ST         DEIHYLSAIEEGNYVIAQANSNLDDEGHFVEDLVTCRSKGESSLFSRDQVDYMDVSTQQV
                ************************:***********************************

RpoB K-12       VSVGASLIPFLEHDDANRALMGANMQRQAVPTLRADKPLVGTGMERAVAVDSGVTAVAKR
RpoB EHEC       VSVGASLIPFLEHDDANRALMGANMQRQAVPTLRADKPLVGTGMERAVAVDSGVTAVAKR
RpoB ST         VSVGASLIPFLEHDDANRALMGANMQRQAVPTLRADKPLVGTGMERAVAVDSGVTAVAKR
                ************************************************************

RpoB K-12       GGVVQYVDASRIVIKVNEDEMYPGEAGIDIYNLTKYTRSNQNTCINQMPCVSLGEPVERG
RpoB EHEC       GGVVQYVDASRIVIKVNEDEMYPGEAGIDIYNLTKYTRSNQNTCINQMPCVSLGEPVERG
RpoB ST         GGTVQYVDASRIVIKVNEDEMYPGEAGIDIYNLTKYTRSNQNTCINQMPCVSLGEPVERG
                **.*********************************************************

RpoB K-12       DVLADGPSTDLGELALGQNMRVAFMPWNGYNFEDSILVSERVVQEDRFTTIHIQELACVS
RpoB EHEC       DVLADGPSTDLGELALGQNMRVAFMPWNGYNFEDSILVSERVVQEDRFTTIHIQELACVS
RpoB ST         DVLADGPSTDLGELALGQNMRVAFMPWNGYNFEDSILVSERVVQEDRFTTIHIQELACVS
                ************************************************************

RpoB K-12       RDTKLGPEEITADIPNVGEAALSKLDESGIVYIGAEVTGGDILVGKVTPKGETQLTPEEK
RpoB EHEC       RDTKLGPEEITADIPNVGEAALSKLDESGIVYIGAEVTGGDILVGKVTPKGETQLTPEEK
RpoB ST         RDTKLGPEEITADIPNVGEAALSKLDESGIVYIGAEVTGGDILVGKVTPKGETQLTPEEK
                ************************************************************

RpoB K-12       LLRAIFGEKASDVKDSSLRVPNGVSGTVIDVQVFTRDGVEKDKRALEIEEMQLKQAKKDL
RpoB EHEC       LLRAIFGEKASDVKDSSLRVPNGVSGTVIDVQVFTRDGVEKDKRALEIEEMQLKQAKKDL
RpoB ST         LLRAIFGEKASDVKDSSLRVPNGVSGTVIDVQVFTRDGVEKDKRALEIEEMQLKQAKKDL
                ************************************************************

RpoB K-12       SEELQILEAGLFSRIRAVLVAGGVEAEKLDKLPRDRWLELGLTDEEKQNQLEQLAEQYDE
RpoB EHEC       SEELQILEAGLFSRIRAVLVAGGVEAEKLDKLPRDRWLELGLTDEEKQNQLEQLAEQYDE
RpoB ST         SEELQILEAGLFSRIRAVLVSSGVEAEKLDKLPRDRWLELGLTDEEKQNQLEQLAEQYDE
                ********************:.**************************************

RpoB K-12       LKHEFEKKLEAKRRKITQGDDLAPGVLKIVKVYLAVKRRIQPGDKMAGRHGNKGVISKIN
RpoB EHEC       LKHEFEKKLEAKRRKITQGDDLAPGVLKIVKVYLAVKRRIQPGDKMAGRHGNKGVISKIN
RpoB ST         LKHEFEKKLEAKRRKITQGDDLAPGVLKIVKVYLAVKRRIQPGDKMAGRHGNKGVISKIN
                ************************************************************

RpoB K-12       PIEDMPYDENGTPVDIVLNPLGVPSRMNIGQILETHLGMAAKGIGDKINAMLKQQQEVAK
RpoB EHEC       PIEDMPYDENGTPVDIVLNPLGVPSRMNIGQILETHLGMAAKGIGDKINAMLKQQQEVAK
RpoB ST         PIEDMPYDENGTPVDIVLNPLGVPSRMNIGQILETHLGMAAKGIGDKINAMLKQQQEVAK
                ************************************************************
```

```
RpoB K-12       LREFIQRAYDLGADVRQKVDLSTFSDEEVMRLAENLRKGMPIATPVFDGAKEAEIKELLK
RpoB EHEC       LREFIQRAYDLGADVRQKVDLSTFSDEEVMRLAENLRKGMPIATPVFDGAKEAEIKELLK
RpoB ST         LREFIQRAYDLGADVRQKVDLSTFSDDEVLRLAENLRKGMPIATPVFDGAKEAEIKELLK
                **************************:**:******************************

RpoB K-12       LGDLPTSGQIRLYDGRTGEQFERPVTVGYMYMLKLNHLVDDKMHARSTGSYSLVTQQPLG
RpoB EHEC       LGDLPTSGQIRLYDGRTGEQFERPVTVGYMYMLKLNHLVDDKMHARSTGSYSLVTQQPLG
RpoB ST         LGDLPTSGQITLFDGRTGEQFERPVTVGYMYMLKLNHLVDDKMHARSTGSYSLVTQQPLG
                ********** *:***********************************************

RpoB K-12       GKAQFGGQRFGEMEVWALEAYGAAYTLQEMLTVKSDDVNGRTKMYKNIVDGNHQMEPGMP
RpoB EHEC       GKAQFGGQRFGEMEVWALEAYGAAYTLQEMLTVKSDDVNGRTKMYKNIVDGNHQMEPGMP
RpoB ST         GKAQFGGQRFGEMEVWALEAYGAAYTLQEMLTVKSDDVNGRTKMYKNIVDGNHQMEPGMP
                ************************************************************

RpoB K-12       ESFNVLLKEIRSLGINIELEDE
RpoB EHEC       ESFNVLLKEIRSLGINIELEDE
RpoB ST         ESFNVLLKEIRSLGINIELEDE
                **********************
```

F RNA polymerase β' subunit/ RpoC

```
RpoC K-12       MKDLLKFLKAQTKTEEFDAIKIALASPDMIRSWSFGEVKKPETINYRTFKPERDGLFCAR
RpoC EHEC       MKDLLKFLKAQTKTEEFDAIKIALASPDMIRSWSFGEVKKPETINYRTFKPERDGLFCAR
RpoC ST         MKDLLKFLKAQTKTEEFDAIKIALASPDMIRSWSFGEVKKPETINYRTFKPERDGLFCAR
                ************************************************************

RpoC K-12       IFGPVKDYECLCGKYKRLKHRGVICEKCGVEVTQTKVRRERMGHIELASPTAHIWFLKSL
RpoC EHEC       IFGPVKDYECLCGKYKRLKHRGVICEKCGVEVTQTKVRRERMGHIELASPTAHIWFLKSL
RpoC ST         IFGPVKDYECLCGKYKRLKHRGVICEKCGVEVTQTKVRRERMGHIELASPTAHIWFLKSL
                ************************************************************

RpoC K-12       PSRIGLLLDMPLRDIERVLYFESYVVIEGGMTNLERQQILTEEQYLDALEEFGDEFDAKM
RpoC EHEC       PSRIGLLLDMPLRDIERVLYFESYVVIEGGMTNLERQQILTEEQYLDALEEFGDEFDAKM
RpoC ST         PSRIGLLLDMPLRDIERVLYFESYVVIEGGMTNLERQQILTEEQYLDALEEFGDEFDAKM
                ************************************************************

RpoC K-12       GAEAIQALLKSMDLEQECEQLREELNETNSETKRKKLTKRIKLLEAFVQSGNKPEWMILT
RpoC EHEC       GAEAIQALLKSMDLEQECEQLREELNETNSETKRKKLTKRIKLLEAFVQSGNKPEWMILT
RpoC ST         GAEAIQALLKSMDLEQECETLREELNETNSETKRKKLTKRIKLLEAFVQSGNKPEWMILT
                ******************* ****************************************

RpoC K-12       VLPVLPPDLRPLVPLDGGRFATSDLNDLYRRVINRNNRLKRLLDLAAPDIIVRNEKRMLQ
RpoC EHEC       VLPVLPPDLRPLVPLDGGRFATSDLNDLYRRVINRNNRLKRLLDLAAPDIIVRNEKRMLQ
RpoC ST         VLPVLPPDLRPLVPLDGGRFATSDLNDLYRRVINRNNRLKRLLDLAAPDIIVRNEKRMLQ
                ************************************************************

RpoC K-12       EAVDALLDNGRRGRAITGSNKRPLKSLADMIKGKQGRFRQNLLGKRVDYSGRSVITVGPY
RpoC EHEC       EAVDALLDNGRRGRAITGSNKRPLKSLADMIKGKQGRFRQNLLGKRVDYSGRSVITVGPY
RpoC ST         EAVDALLDNGRRGRAITGSNKRPLKSLADMIKGKQGRFRQNLLGKRVDYSGRSVITVGPY
                ************************************************************

RpoC K-12       LRLHQCGLPKKMALELFKPFIYGKLELRGLATTIKAAKKMVEREEAVVWDILDEVIREHP
RpoC EHEC       LRLHQCGLPKKMALELFKPFIYGKLELRGLATTIKAAKKMVEREEAVVWDILDEVIREHP
RpoC ST         LRLHQCGLPKKMALELFKPFIYGKLELRGLATTIKAAKKMVEREEAVVWDILDEVIREHP
                ************************************************************

RpoC K-12       VLLNRAPTLHRLGIQAFEPVLIEGKAIQLHPLVCAAYNADFDGDQMAVHVPLTLEAQLEA
RpoC EHEC       VLLNRAPTLHRLGIQAFEPVLIEGKAIQLHPLVCAAYNADFDGDQMAVHVPLTLEAQLEA
RpoC ST         VLLNRAPTLHRLGIQAFEPVLIEGKAIQLHPLVCAAYNADFDGDQMAVHVPLTLEAQLEA
                ************************************************************
```

```
RpoC K-12       RALMMSTNNILSPANGEPIIVPSQDVVLGLYYMTRDCVNAKGEGMVLTGPKEAERLYRSG
RpoC EHEC       RALMMSTNNILSPANGEPIIVPSQDVVLGLYYMTRDCVNAKGEGMVLTGPKEAERLYRSG
RpoC ST         RALMMSTNNILSPANGEPIIVPSQDVVLGLYYMTRDCVNAKGEGMVLTGPKEAERIYRAG
                *******************************************************:**:*

RpoC K-12       LASLHARVKVRITEYEKDANGELVAKTSLKDTTVGRAILWMIVPKGLPYSIVNQALGKKA
RpoC EHEC       LASLHARVKVRITEYEKDANGELVAKTSLKDTTVGRAILWMIVPKGLPYSIVNQALGKKA
RpoC ST         LASLHARVKVRITEYEKDENGEFVAHTSLKDTTVGRAILWMIVPKGLPFSIVNQALGKKA
                ****************** ***:**:**********************:***********

RpoC K-12       ISKMLNTCYRILGLKPTVIFADQIMYTGFAYAARSGASVGIDDMVIPEKKHEIISEAEAE
RpoC EHEC       ISKMLNTCYRILGLKPTVIFADQIMYTGFAYAARSGASVGIDDMVIPEKKHEIISEAEAE
RpoC ST         ISKMLNTCYRILGLKPTVIFADQTMYTGFAYAARSGASVGIDDMVIPEKKHEIISEAEAE
                ********************** *************************************

RpoC K-12       VAEIQEQFQSGLVTAGERYNKVIDIWAAANDRVSKAMMDNLQTETVINRDGQEEKQVSFN
RpoC EHEC       VAEIQEQFQSGLVTAGERYNKVIDIWAAANDRVSKAMMDNLQTETVINRDGQEEKQVSFN
RpoC ST         VAEIQEQFQSGLVTAGERYNKVIDIWAAANDRVSKAMMDNLQTETVINRDGQEEQQVSFN
                ******************************************************:*****

RpoC K-12       SIYMMADSGARGSAAQIRQLAGMRGLMAKPDGSIIETPITANFREGLNVLQYFISTHGAR
RpoC EHEC       SIYMMADSGARGSAAQIRQLAGMRGLMAKPDGSIIETPITANFREGLNVLQYFISTHGAR
RpoC ST         SIYMMADSGARGSAAQIRQLAGMRGLMAKPDGSIIETPITANFREGLNVLQYFISTHGAR
                ************************************************************

RpoC K-12       KGLADTALKTANSGYLTRRLVDVAQDLVVTEDDCGTHEGIMMTPVIEGGDVKEPLRDRVL
RpoC EHEC       KGLADTALKTANSGYLTRRLVDVAQDLVVTEDDCGTHEGIMMTPVIEGGDVKEPLRDRVL
RpoC ST         KGLADTALKTANSGYLTRRLVDVAQDLVVTEDDCGTHEGILMTPVIEGGDVKEPLRDRVL
                ****************************************:*******************

RpoC K-12       GRVTAEDVLKPGTADILVPRNTLLHEQWCDLLEENSVDAVKVRSVVSCDTDFGVCAHCYG
RpoC EHEC       GRVTAEDVLKPGTADILVPRNTLLHEQWCDLLEENSVDAVKVRSVVSCDTDFGVCAHCYG
RpoC ST         GRVTAEDVLKPGTADILVPRNTLLHEQWCDLLEANSVDAVKVRSVVSCDTDFGVCAHCYG
                ********************************* **************************

RpoC K-12       RDLARGHIINKGEAIGVIAAQSIGEPGTQLTMRTFHIGGAASRAAAESSIQVKNKGSIKL
RpoC EHEC       RDLARGHIINKGEAIGVIAAQSIGEPGTQLTMRTFHIGGAASRAAAESSIQVKNKGSIKL
RpoC ST         RDLARGHIINKGEAIGVIAAQSIGEPGTQLTMRTFHIGGAASRAAAESSIQVKNKGSIKL
                ************************************************************

RpoC K-12       SNVKSVVNSSGKLVITSRNTELKLIDEFGRTKESYKVPYGAVLAKGDGEQVAGGETVANW
RpoC EHEC       SNVKSVVNSSGKLVITSRNTELKLIDEFGRTKESYKVPYGAVLAKGDGEQVAGGETVANW
RpoC ST         SNVKSVVNSSGKLVITSRNTELKLIDEFGRTKESYKVPYGAVMAKGDGEQVAGGETVANW
                ******************************************:*****************

RpoC K-12       DPHTMPVITEVSGFVRFTDMIDGQTITRQTDELTGLSSLVVLDSAERTAGGKDLRPALKI
RpoC EHEC       DPHTMPVITEVSGFVRFTDMIDGQTITRQTDELTGLSSLVVLDSAERTAGGKDLRPALKI
RpoC ST         DPHTMPVITEVSGFIRFTDMIDGQTITRQTDELTGLSSLVVLDSAERTTGGKDLRPALKI
                **************:*********************************:***********

RpoC K-12       VDAQGNDVLIPGTDMPAQYFLPGKAIVQLEDGVQISSGDTLARIPQESGGTKDITGGLPR
RpoC EHEC       VDAQGNDVLIPGTDMPAQYFLPGKAIVQLEDGVQISSGDTLARIPQESGGTKDITGGLPR
RpoC ST         VDAQGNDVLIPGTDMPAQYFLPGKAIVQLEDGVQISSGDTLARIPQESGGTKDITGGLPR
                ************************************************************

RpoC K-12       VADLFEARRPKEPAILAEISGIVSFGKETKGKRRLVITPVDGSDPYEEMIPKWRQLNVFE
RpoC EHEC       VADLFEARRPKEPAILAEISGIVSFGKETKGKRRLVITPVDGSDPYEEMIPKWRQLNVFE
RpoC ST         VADLFEARRPKEPAILAEIAGIVSFGKETKGKRRLVITPVDGSDPYEEMIPKWRQLNVFE
                ********************:***************************************

RpoC K-12       GERVERGDVISDGPEAPHDILRLRGVHAVTRYIVNEVQDVYRLQGVKINDKHIEVIVRQM
RpoC EHEC       GERVERGDVISDGPEAPHDILRLRGVHAVTRYIVNEVQDVYRLQGVKINDKHIEVIVRQM
RpoC ST         GERVERGDVISDGPEAPHDILRLRGVHAVTRYIVNEVQDVYRLQGVKINDKHIEVIVRQM
                ************************************************************
```

```
RpoC K-12       LRKATIVNAGSSDFLEGEQVEYSRVKIANRELEANGKVGATYSRDLLGITKASLATESFI
RpoC EHEC       LRKATIVNAGSSDFLEGEQVEYSRVKIANRELEANGKVGATYSRDLLGITKASLATESFI
RpoC ST         LRKATIESAGSSDFLEGEQVEYSRVKIANRELEANGKVGATFSRDLLGITKASLATESFI
                ****** .*********************************:******************

RpoC K-12       SAASFQETTRVLTEAAVAGKRDELRGLKENVIVGRLIPAGTGYAYHQDRMRRRAAGEAPA
RpoC EHEC       SAASFQETTRVLTEAAVAGKRDELRGLKENVIVGRLIPAGTGYAYHQDRMRRRAAGEAPA
RpoC ST         SAASFQETTRVLTEAAVAGKRDELRGLKENVIVGRLIPAGTGYAYHQDRMRRRAAGEQPA
                ********************************************************* **

RpoC K-12       APQVTAEDASASLAELLNAGLGGSDNE
RpoC EHEC       APQVTAEDASASLAELLNAGLGGSDNE
RpoC ST         TPQVTAEDASASLAELLNAGLGGSDNE
                :**************************
```

G RNA polymerase σ^{70} subunit/ RpoD

```
RpoD K-12       MEQNPQSQLKLLVTRGKEQGYLTYAEVNDHLPEDIVDSDQIEDIIQMINDMGIQVMEEAP
RpoD EHEC       MEQNPQSQLKLLVTRGKEQGYLTYAEVNDHLPEDIVDSDQIEDIIQMINDMGIQVMEEAP
RpoD ST         MEQNPQSQLKLLVTRGKEQGYLTYAEVNDHLPEDIVDSDQIEDIIQMINDMGIQVMEEAP
                ************************************************************

RpoD K-12       DADDLMLAEN--TADEDAAEAAAQVLSSVESEIGRTTDPVRMYMREMGTVELLTREGEID
RpoD EHEC       DADDLMLAEN--TADEDAAEAAAQVLSSVESEIGRTTDPVRMYMREMGTVELLTREGEID
RpoD ST         DADDLLLAENTTSTDEDAEEAAAQVLSSVESEIGRTTDPVRMYMREMGTVELLTREGEID
                *****:****  ::**** *****************************************

RpoD K-12       IAKRIEDGINQVQCSVAEYPEAITYLLEQYDRVEAEEARLSDLITGFVDPNAEEDLAPTA
RpoD EHEC       IAKRIEDGINQVQCSVAEYPEAITYLLEQYDRVEAEEARLSDLITGFVDPNAEEDLAPTA
RpoD ST         IAKRIEDGINQVQCSVAEYPEAITYLLEQYDRVEAEEARLSDLITGFVDPNAEEEMAPTA
                ******************************************************::****

RpoD K-12       THVGSELSQEDLDDDEDEDEEDGDDDSADDDNSIDPELAREKFAELRAQYVVTRDTIKAK
RpoD EHEC       THVGSELSQEDLDDDEDEDEEDGDDDSADDDNSIDPELAREKFAELRAQYVVTRDTIKAK
RpoD ST         THVGSELSQEDLDDDEDEDEEDGDDDAADDDNSIDPELAREKFAELRAQYVVTRDTIKAK
                **************************:*********************************

RpoD K-12       GRSHATAQEEILKLSEVFKQFRLVPKQFDYLVNSMRVMMDRVRTQERLIMKLCVEQCKMP
RpoD EHEC       GRSHAAAQEEILKLSEVFKQFRLVPKQFDYLVNSMRVMMDRVRTQERLIMKLCVEQCKMP
RpoD ST         GRSHAAAQEEILKLSEVFKQFRLVPKQFDYLVNSMRVMMDRVRTQERLIMKLCVEQCKMP
                *****:******************************************************

RpoD K-12       KKNFITLFTGNETSDTWFNAAIAMNKPWSEKLHDVSEEVHRALQKLQQIEEETGLTIEQV
RpoD EHEC       KKNFITLFTGNETSDTWFNAAIAMNKPWSEKLHDVSEEVHRALQKLQQIEEETGLTIEQV
RpoD ST         KKNFITLFTGNETSETWFNAAIAMNKPWSEKLHDVAEEVQRCLQKLRQIEEETGLTIEQV
                **************:********************:***:*.****:*************

RpoD K-12       KDINRRMSIGEAKARRAKKEMVEANLRLVISIAKKYTNRGLQFLDLIQEGNIGLMKAVDK
RpoD EHEC       KDINRRMSIGEAKARRAKKEMVEANLRLVISIAKKYTNRGLQFLDLIQEGNIGLMKAVDK
RpoD ST         KDINRRMSIGEAKARRAKKEMVEANLRLVISIAKKYTNRGLQFLDLIQEGNIGLMKAVDK
                ************************************************************

RpoD K-12       FEYRRGYKFSTYATWWIRQAITRSIADQARTIRIPVHMIETINKLNRISRQMLQEMGREP
RpoD EHEC       FEYRRGYKFSTYATWWIRQAITRSIADQARTIRIPVHMIETINKLNRISRQMLQEMGREP
RpoD ST         FEYRRGYKFSTYATWWIRQAITRSIADQARTIRIPVHMIETINKLNRISRQMLQEMGREP
                ************************************************************

RpoD K-12       TPEELAERMLMPEDKIRKVLKIAKEPISMETPIGDDEDSHLGDFIEDTTLELPLDSATTE
RpoD EHEC       TPEELAERMLMPEDKIRKVLKIAKEPISMETPIGDDEDSHLGDFIEDTTLELPLDSATTE
RpoD ST         TPEELAERMLMPEDKIRKVLKIAKEPISMETPIGDDEDSHLGDFIEDTTLELPLDSATTE
                ************************************************************
```

```
RpoD K-12       SLRAATHDVLAGLTAREAKVLRMRFGIDMNTDYTLEEVGKQFDVTRERIRQIEAKALRKL
RpoD EHEC       SLRAATHDVLAGLTAREAKVLRMRFGIDMNTDHTLEEVGKQFDVTRERIRQIEAKALRKL
RpoD ST         SLRAATHDVLAGLTAREAKVLRMRFGIDMNTDHTLEEVGKQFDVTRERIRQIEAKALRKL
                ********************************:***************************

RpoD K-12       RHPSRSEVLRSFLDD
RpoD EHEC       RHPSRSEVLRSFLDD
RpoD ST         RHPSRSEVLRSFLDD
                ***************
```

H RNA polymerase ω subunit/ RpoZ

```
RpoZ K-12       MARVTVQDAVEKIGNRFDLVLVAARRARQMQVGGKDPLVPEENDKTTVIALREIEEGLIN
RpoZ ST         MARVTVQDAVEKIGNRFDLVLVAARRARQMQVGGKDPLVPEENDKTTVIALREIEEGLIN
RpoZ EHEC       MARVTVQDAVEKIGNRFDLVLVAARRARQMQVGGKDPLVPEENDKTTVIALREIEEGLIN
                ************************************************************

RpoZ K-12       NQILDVRERQEQQEQEAAELQAVTAIAEGRR
RpoZ ST         NQILDVRERQEQQEQEAAELQAVTAIAEGRR
RpoZ EHEC       NQILDVRERQEQQEQEAAELQAVTAIAEGRR
                *******************************
```

Figure S3.

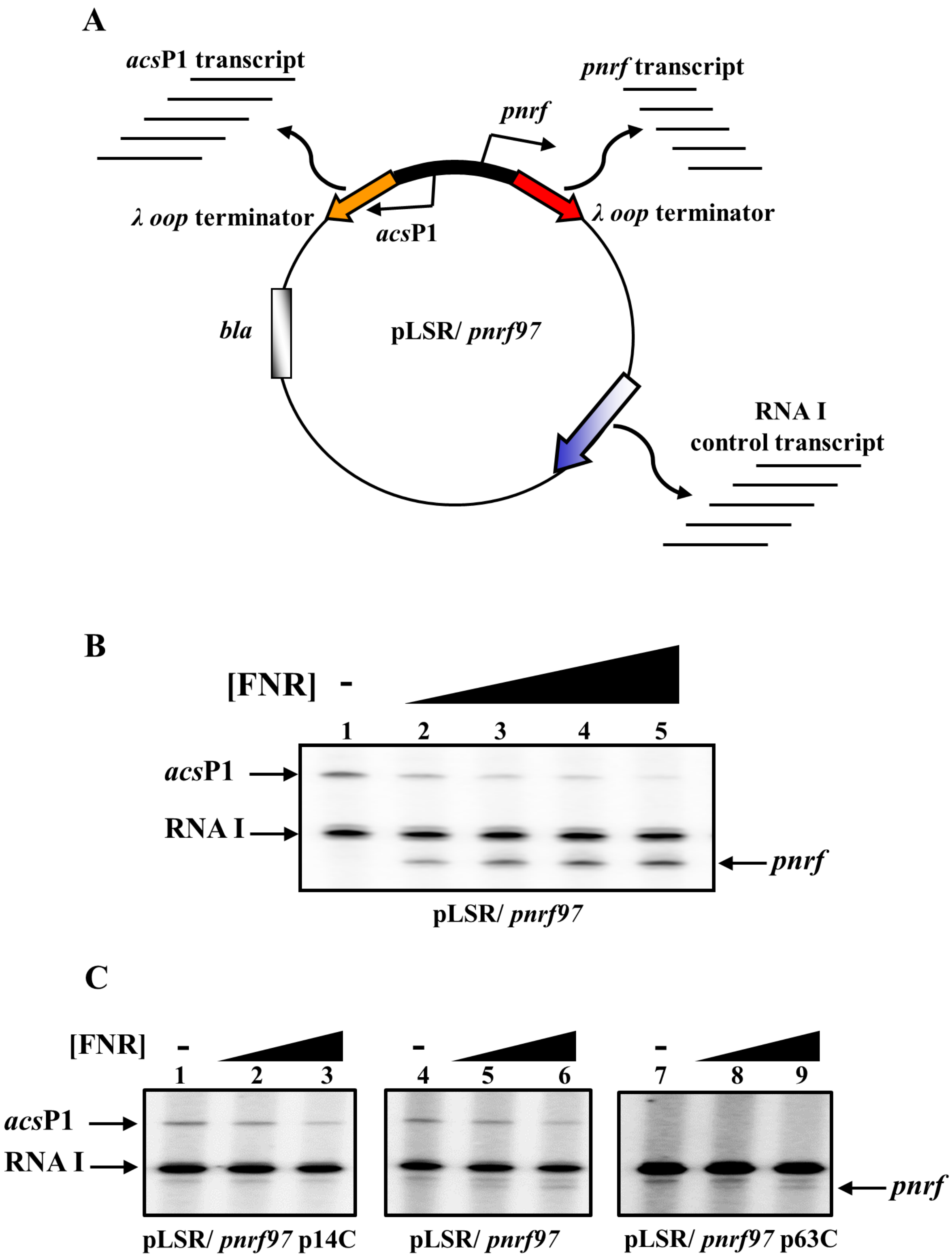
A
acsP1 transcript
pnrf transcript
pnrf
λ oop terminator
acsP1
λ oop terminator
bla
pLSR/ pnrf97
RNA I
control transcript
B
[FNR]
-
1
2
3
4
5
acsP1
RNA I
pnrf
pLSR/ pnrf97
C
[FNR]
-
-
-
1
2
3
4
5
6
7
8
9
acsP1
RNA I
pnrf
pLSR/ pnrf97 p14C
pLSR/ pnrf97
pLSR/ pnrf97 p63C

Figure S4.

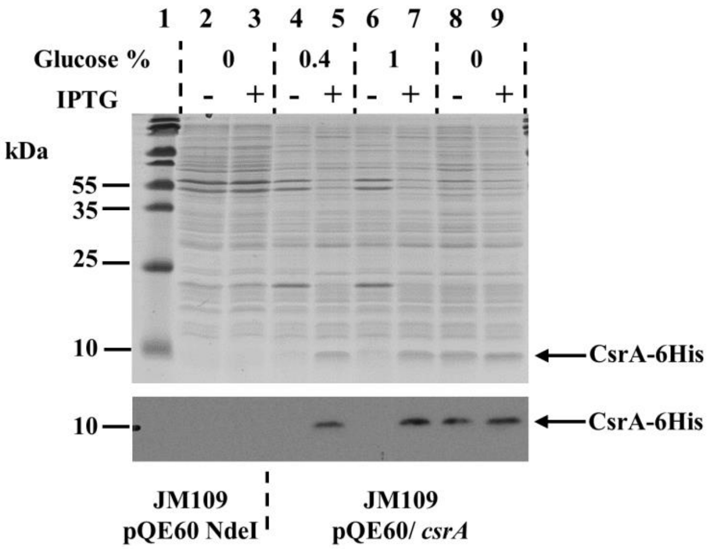
1 2 3 4 5 6 7 8 9
Glucose %
0
0.4
1
0
IPTG
- + - + - + - +
kDa
55
35
25
10
CsrA-6His
10
CsrA-6His
JM109
pQE60 NdeI
JM109
pQE60/ *csrA*

Figure S5.

A

```
                              FNR                             -10       +1
MG1655          CACTTACAATTGATTAAAGACAACATTCACAGTGTGGTTATTTGTTACACATAGGGGCGA
TW14359         CACTTACAATTGATTAAAGACAACATTCACAGTGTGGTTATTTGTTACACTTAGGGGCGA
TW14588         CACTTACAATTGATTAAAGACAACATTCACAGTGTGGTTATTTGTTACACTTAGGGGCGA
Sakai           CACTTACAATTGATTAAAGACAACATTCACAGTGTGGTTATTTGTTACACTTAGGGGCGA
G5101           CACTTACAATTGATTAAAGACAACATTCACAGTGTGGTTATTTGTTACACTTAGGGGCGA
EC4401          CACTTACAATTGATTAAAGACAACATTCACAGTGTGGTTATTTGTTACACTTAGGGGCGA
EC4486          CACTTACAATTGATTAAAGACAACATTCACAGTGTGGTTATTTGTTACACTTAGGGGCGA
EC4501          CACTTACAATTGATTAAAGACAACATTCACAGTGTGGTTATTTGTTACACTTAGGGGCGA
EC508           CACTTACAATTGATTAAAGACAACATTCACAGTGTGGTTATTTGTTACACTTAGGGGCGA
EC869           CACTTACAATTGATTAAAGACAACATTCACAGTGTGGTTATTTGTTACACTTAGGGGCGA
EC4206          CACTTACAATTGATTAAAGACAACATTCACAGTGTGGTTATTTGTTACACTTAGGGGCGA
EC4196          CACTTACAATTGATTAAAGACAACATTCACAGTGTGGTTATTTGTTACACTTAGGGGCGA
EC4115          CACTTACAATTGATTAAAGACAACATTCACAGTGTGGTTATTTGTTACACTTAGGGGCGA
EC4113          CACTTACAATTGATTAAAGACAACATTCACAGTGTGGTTATTTGTTACACTTAGGGGCGA
EC4076          CACTTACAATTGATTAAAGACAACATTCACAGTGTGGTTATTTGTTACACTTAGGGGCGA
EC4045          CACTTACAATTGATTAAAGACAACATTCACAGTGTGGTTATTTGTTACACTTAGGGGCGA
EC4024          CACTTACAATTGATTAAAGACAACATTCACAGTGTGGTTATTTGTTACACTTAGGGGCGA
EC4009          CACTTACAATTGATTAAAGACAACATTCACAGTGTGGTTATTTGTTACACTTAGGGGCGA
EC1212          CACTTACAATTGATTAAAGACAACATTCACAGTGTGGTTATTTGTTACACTTAGGGGCGA
1044            CACTTACAATTGATTAAAGACAACATTCACAGTGTGGTTATTTGTTACACTTAGGGGCGA
1125            CACTTACAATTGATTAAAGACAACATTCACAGTGTGGTTATTTGTTACACTTAGGGGCGA
EDL933          CACTTACAATTGATTAAAGACAACATTCACAGTGTGGTTATTTGTTACACTTAGGGGCGA
                ************************************************** *********
                              FNR                             -10       +1
```

B

```
                              FNR                             -10       +1
MG1655          CACTTACAATTGATTAAAGACAACATTCACAGTGTGGTTATTTGTTACACATAGGGGCGA
CB9615          CACTTACAATTGATTAAAGACAACATTCACAGTGTGGTTATTTGTTACACATAGGGGCGA
3256-97         CACTTACAATTGATTAAAGACAACATTCACAGTGTGGTTATTTGTTACACATAGGGGCGA
USDA5905        CACTTACAATTGATTAAAGACAACATTCACAGTGTGGTTATTTGTTACACATAGGGGCGA
493-89          CACTTACAATTGATTAAAGACAACATTCACAGTGTGGTTATTTGTTACACATAGGGGCGA
H2687           CACTTACAATTGATTAAAGACAACATTCACAGTGTGGTTATTTGTTACACATAGGGGCGA
G5101           CACTTACAATTGATTAAAGACAACATTCACAGTGTGGTTATTTGTTACACTTAGGGGCGA
Sakai           CACTTACAATTGATTAAAGACAACATTCACAGTGTGGTTATTTGTTACACTTAGGGGCGA
EDL933          CACTTACAATTGATTAAAGACAACATTCACAGTGTGGTTATTTGTTACACTTAGGGGCGA
TW14359         CACTTACAATTGATTAAAGACAACATTCACAGTGTGGTTATTTGTTACACTTAGGGGCGA
EC4115          CACTTACAATTGATTAAAGACAACATTCACAGTGTGGTTATTTGTTACACTTAGGGGCGA
                ************************************************** *********
                              FNR                             -10       +1
```

Figure S6.

```
                              FNR                                -10          -3  +1   +4
Typhimurium         TTACAATTGATTAAAGACAACATTTTAAGTGTGGTTATTTGTTACACATAGAGGAGATCA
Heidelberg          TTACAATTGATTAAAGACAACATTTTAAGTGTGGTTATTTGTTACACATAGAGGAGATCA
Gallinarum          TTACAATTGATTAAAGACAACATTTTAAGTGTGGTTATTTGTTACACATAGAGGAGATCA
Enteritidis         TTACAATTGATTAAAGACAACATTTTAAGTGTGGTTATTTGTTACACATAGAGGAGATCA
Choleraesuis        TTACAATTGATTAAAGACAACATTTTAAGTGTGGTTATTTGTTACACATAGAGGAGATCA
Typhi               TTACAATTGATTAAAGACAACATTTTGAGTGTGGTTATTTGTTACACATAGAGGAGATCA
Paratyphi A         TTACAATTGATTAAAGACAACATTTTGAGTGTGGTTATTTGTTACACATAGAGGAGATCA
Paratyphi B         TTACAATTGATTAAAGACAACATTTTAAGTGTGGTTATTTGTTACACATAGAGGAGATCA
Paratyphi C         TTACAATTGATTAAAGACAACATTTTAAGTGTGGTTATTTGTTACACATAGAGGAGATCA
Schwarzengrund      TTACAATTGATTAAAGACAACATTTTAAGTGTGGTTATTTGTTACACATAGAGGAGATCA
Dublin              TTACAATTGATTAAAGACAACATTTTAAGTGTGGTTATTTGTTACACATAGAGGAGAGCA
Newport             TTACAATTGATTAAAGACAACATTTTAAGTGTGGTTATTTGTTACACATAGAGGAGAGCA
E. coli K-12        TTACAATTGATTAAAGACAACATTCACAGTGTGGTTATTTGTTACACATAGGGGCGAGCA
                    ************************   ************************ ** ** **
                              FNR                                -10          -3  +1   +4
```
